# Aneuploidy of specific chromosomes is beneficial to cells lacking spindle checkpoint protein Bub3

**DOI:** 10.1371/journal.pgen.1011576

**Published:** 2025-02-04

**Authors:** Pallavi Gadgil, Olivia Ballew, Timothy J. Sullivan, Soni Lacefield

**Affiliations:** 1 Department of Biochemistry and Cell Biology, Dartmouth College Geisel School of Medicine, Hanover, New Hampshire, United States of America; 2 Department of Biology, Indiana University Bloomington, Bloomington, Indiana, United States of America; 3 Department of Biomedical Data Science, Dartmouth College Geisel School of Medicine, Hanover, New Hampshire, United States of America; Stowers Institute for Medical Research, UNITED STATES OF AMERICA

## Abstract

Aneuploidy typically poses challenges for cell survival and growth. However, recent studies have identified exceptions where aneuploidy is beneficial for cells with mutations in certain regulatory genes. Our research reveals that cells lacking the spindle checkpoint gene *BUB3* exhibit aneuploidy of select chromosomes. While the spindle checkpoint is not essential in budding yeast, the loss of *BUB3* and *BUB1* increases the probability of chromosome missegregation compared to wildtype cells. Contrary to the prevailing assumption that the aneuploid cells would be outcompeted due to growth defects, our findings demonstrate that *bub3*Δ cells consistently maintained aneuploidy of specific chromosomes over many generations. We investigated whether the persistence of these additional chromosomes in *bub3*Δ cells resulted from the beneficial elevated expression of certain genes, or mere tolerance. We identified several genes involved in chromosome segregation and cell cycle regulation that confer an advantage to Bub3-depleted cells. Overall, our results suggest that the gain of specific genes through aneuploidy may provide a survival advantage to strains with poor chromosome segregation fidelity.

## Author summary

Accurate chromosome segregation is crucial for the proper development of all living organisms. Errors in chromosome segregation can lead to aneuploidy, characterized by an abnormal number of chromosomes, which generally impairs cell survival and growth. However, under certain stress conditions, such as in various cancers, cells with specific mutations and extra copies of advantageous chromosomes exhibit improved survival and proliferation. In our study, we discovered that cells lacking the spindle checkpoint protein Bub3 became aneuploid, retaining specific chromosomes. This finding was unexpected because although *bub3*Δ cells have a higher rate of chromosome mis-segregation, they were not thought to maintain an aneuploid karyotype. We investigated whether the increased copy number of specific genes on these acquired chromosomes offered a benefit to Bub3-deficient cells. Our results revealed that several genes involved in chromosome segregation and cell cycle regulation prevented the gain of chromosomes upon Bub3-depletion, suggesting that these genes confer an advantage by reducing chromosome missegregation. Overall, our study demonstrates that cells lacking Bub3 selectively retain specific chromosomes to increase the copy number of genes that promote proper chromosome segregation.

## Introduction

Errors in chromosome segregation can give rise to aneuploid cells, which have an abnormal number of chromosomes. Aneuploidy can be deleterious to cells by causing an imbalance in protein expression and proteotoxic stress, which affects both survival and growth [1–4]. Aneuploidy can be particularly detrimental during the development of multicellular organisms. However, there are conditions where aneuploidy provides a benefit to cells, allowing them to grow and divide during stress [5,6]. For example, many pathogenic fungi are aneuploid and chromosome gain can provide drug resistance during infection by increasing the copy number of drug efflux transporters [7]. Similarly, most solid tumors are aneuploid, which likely contributes to cancer progression [8,9]. Finally, although aneuploidy causes growth defects in most budding yeast lab strains, a gain of chromosomes can be beneficial to cells growing in stressful conditions, allowing rapid adaptive evolution [3,4,10]. Furthermore, cells with mutations in some regulatory genes may benefit from aneuploidy [9,11–16]. Therefore, while a high fidelity of chromosome segregation is crucial for survival, allowing occasional errors in segregation may provide a mechanism for adaptation to stressful conditions.

Faithful chromosome segregation during mitosis depends on establishing bioriented kinetochore-microtubule attachments, in which the two sister chromatid kinetochores attach to microtubules emanating from opposite spindle poles [17]. Initial attachments are often incorrect with both sister kinetochores attached to the same pole. Error correction mechanisms release incorrect attachments, allowing the establishment of bipolar attachments through cycles of release and reattachment. Additionally, the spindle checkpoint delays the cell cycle in the presence of unattached kinetochores to allow additional time for error correction [18].

In budding yeast, the spindle checkpoint is signaled through the action of several non-essential proteins: Mad1, Mad2, Mad3, Bub1, and Bub3 [19–21]. When a kinetochore is unattached, kinetochore protein Spc105/Knl1 becomes phosphorylated, Bub3 and Bub1 bind the kinetochore and recruit Mad1 and Mad2 [19,22]. This interaction ultimately leads to the formation of the diffusible mitotic checkpoint complex (MCC), which consists of Mad2, Mad3, Bub3, and Cdc20 [18,19]. The MCC inhibits the Anaphase Promoting Complex/ Cyclosome (APC/C), a ubiquitin ligase that ubiquitinates proteins and targets them for proteasomal degradation. Inhibition of the APC/C causes cells to arrest at metaphase. Once kinetochores have established bipolar attachments, Spc105/Knl1 is dephosphorylated, the spindle checkpoint proteins are released from the kinetochore, the MCC is disassembled, and anaphase onset ensues.

Although the spindle checkpoint proteins are not essential in budding yeast, *bub1*Δ and *bub3*Δ cells are slow-growing and have a prolonged metaphase, unlike *mad1*Δ, *mad2*Δ and *mad3*Δ cells, which grow similarly to wildtype [20,21,23–25]. Several studies show that Bub3 and Bub1 have additional functions at the kinetochore besides in spindle checkpoint signaling [25]. Our previous work showed that Bub3, but not Bub1, facilitates binding of APC/C and Cdc20 at the kinetochore. Furthermore, both Bub3 and Bub1 help recruit Sgo1 to the kinetochore. Sgo1 is important for the biorientation of sister chromatid kinetochores and serves as a platform to recruit other proteins including the chromosome passenger complex (CPC) [26,27]. The CPC contains Ipl1/Aurora kinase B, which is required for error correction of improper kinetochore-microtubule attachments [17]. Ipl1/Aurora B phosphorylates kinetochore proteins to release attachments that are not under tension. In budding yeast, several redundant pathways recruit the CPC to the kinetochore, and therefore, depletion of CPC components has a more severe phenotype than loss of Bub1 and Bub3 [27–33].

Despite the increased rate of chromosome missegregation in *bub1*Δ and *bub3*Δ cells, the previous assumption was that the aneuploid cells would grow slower than the euploid cells and would be outcompeted in the population [23]. The euploid cells would still have a chromosome mis-segregation phenotype, slowing the overall growth rate of the population in comparison to wildtype because the cells with chromosome loss would die. However, we noticed that *bub1*Δ and *bub3*Δ cells had an abnormal morphology, prompting us to ask if the aneuploid cells did indeed take over the population. Using a whole genome sequencing approach, we found that *bub3*Δ cells quickly acquired additional copies of one or more of five specific chromosomes: I, II, III, VIII, and X. Over generations, the aneuploidy was persistent yet dynamic, switching between those five chromosomes. We asked which genes on the chromosomes may provide a benefit to the *bub3*Δ cells when the copy number was increased. Our results suggest that several genes, including those involved in chromosome segregation and cell cycle regulation, are advantageous to *bub3*Δ cells when gained either individually or in combination. Thus, aneuploidy may be a strategy that allows *bub3*Δ cells to survive and persist despite having lower chromosome segregation fidelity.

## Results

### The loss of *BUB3* shows the gain of specific chromosomes

**Fig 1:**
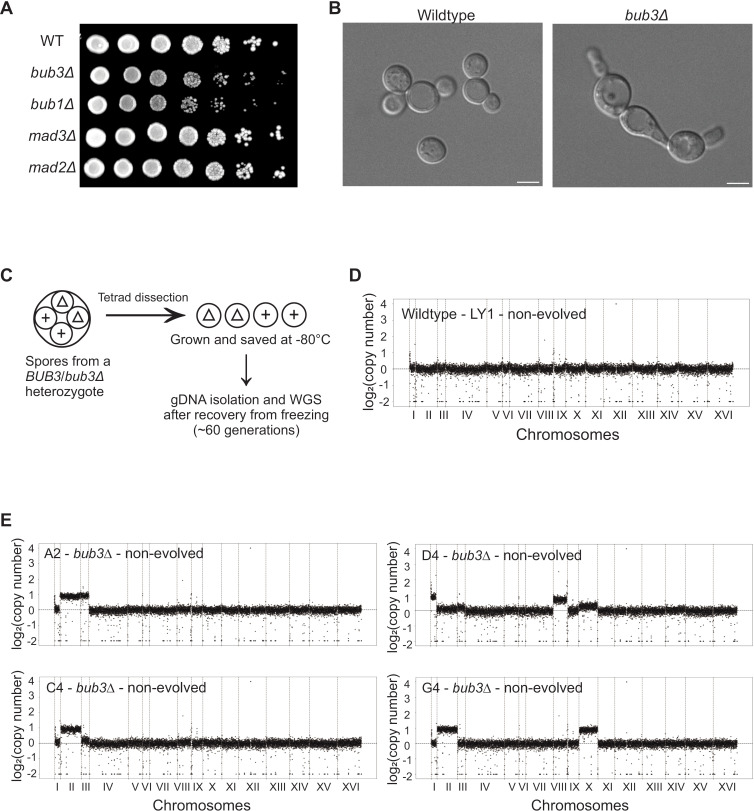
Loss of *BUB3* causes growth, morphological, and chromosome segregation defects. **(A)** Comparison of growth of wildtype and spindle checkpoint deleted strains. Saturated yeast cultures were serially diluted, spotted on YPD plates, and imaged after 40 hours of incubation. **(B)** Representative DIC images of the morphological differences between wildtype and *bub3*Δ cells. Scale bar = 5μm. (**C)** Workflow of obtaining haploids for whole genome sequencing after 60 generations of growth. **(D, E)** The CNV plots of wildtype (D) or *bub3*Δ (E) cells from whole genome sequencing. The x-axis shows the 16 budding yeast chromosomes separated by vertical dotted lines according to size. The y-axis shows log_2_(copy number). The horizontal dotted line delineates 1 chromosome copy. Each increment shows an additional chromosome copy.

Although the spindle checkpoint proteins are not essential in budding yeast, the loss of *bub1*Δ and *bub3*Δ cells have delayed growth, in contrast to *mad1*Δ, *mad2*Δ and *mad3*Δ cells [23,25]. To further characterize the growth delay, we spotted serial dilutions of saturated yeast cultures onto rich media plates. The *bub1*Δ and *bub3*Δ cells showed a growth difference and a significantly longer doubling time when compared to wildtype, *mad2*Δ, and *mad3*Δ cells (Figs 1A, S1A and S1B). There are multiple potential reasons for an extended doubling time. First, we previously showed that *bub3*Δ cells are delayed in metaphase II likely due to slower APC/C activation [25]. Second, the overall doubling time could be slower due to the increased rate of chromosome mis-segregation, with the death of cells that lost a chromosome and slower growth of cells that gained a chromosome. Third, there could be a high percentage of cells that have chromosome gain that took over the population. Cells with extra chromosomes tend to have a growth delay, and therefore, an accumulation of these cells could extend the overall growth rate of the population [4]. These possibilities are not mutually exclusive.

Interestingly, we also observed that *bub1*Δ and *bub3*Δ cells had morphological defects, in which the cells were misshaped, larger, elongated, and sometimes formed chains (Figs 1B–S1C). The abnormal morphology prompted us to investigate whether *bub1*Δ and *bub3*Δ cells were aneuploid or had acquired mutations that affected their growth and morphology. The previous assumption was that although *bub3*Δ cells had an increased probability of chromosome missegregation, the aneuploid cells would be outcompeted by the euploid population because they grow slower. We decided to perform further analysis on *bub3*Δ cells because *bub3*Δ cells had a more severe growth defect than *bub1*Δ cells.

To determine if the observed growth defects of *bub3*Δ cells were due to chromosome copy number variation (CNV), we performed whole genome sequencing of *bub3*Δ cells. To ensure that we started the experiment with a euploid cell, we deleted one copy of *BUB3* in a wildtype diploid to make a *BUB3*/*bub3*Δ heterozygote. We induced meiosis in these diploids and then separated the four resulting haploid spores, of which, two were *BUB3* and two were *bub3*Δ (Fig 1C). From the separated four-spore viable tetrads, the colonies were grown and then kept as frozen stocks. We then recovered the lines and grew them for DNA isolation. This approach minimizes the number of cell division cycles prior to freezing, such that all further experiments are performed on the newly recovered cells from the frozen stocks. We isolated the genomic DNA for whole genome sequencing in these cells that underwent approximately 60 cell cycles.

We sequenced 11 lines of each genotype, choosing some from the same tetrad and some from different tetrads. The lines had very few mutations, none of which were shared among *bub3*Δ lines (S1 Table). As shown in the CNV plots, most of the wildtype strains had one copy of each of the 16 chromosomes (Figs 1D and S2A). Although *BUB3* is haplosufficient, there was one tetrad in which both wildtype strains had an additional set of chromosomes XI and XII, likely due to aneuploidy arising in the mitotic divisions prior to meiosis (S2A Fig).

**Fig 2:**
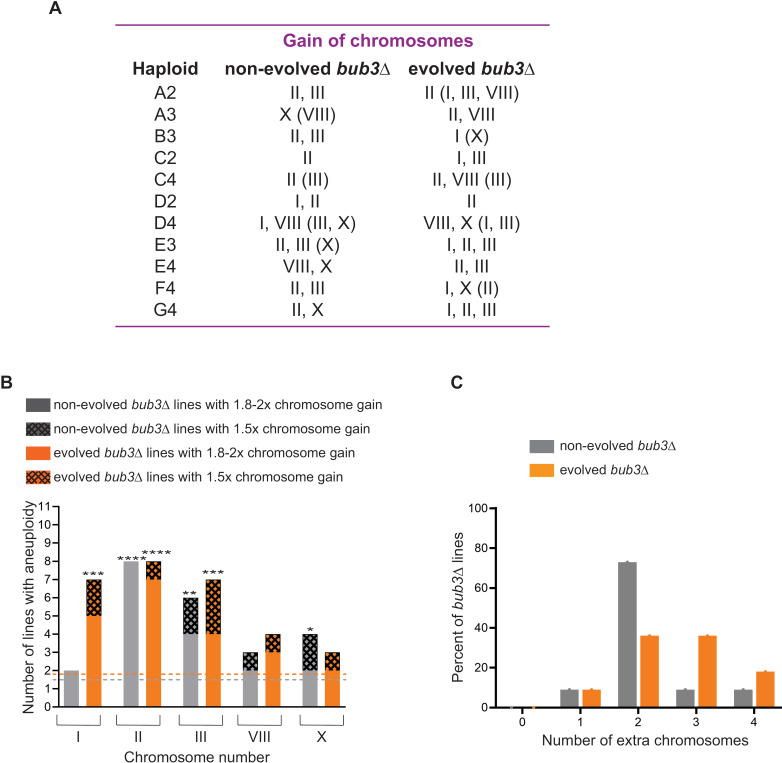
Bub3Δ cells have unstable karyotypes with a chromosome gain bias. (A) Table of the specific chromosomes gained in the non-evolved and evolved bub3Δ lines obtained from whole genome sequencing, in which the population showed a 1.8 to 2-fold increase in that chromosome. In parentheses are the chromosomes that show a 1.5-fold increase in the population. (B) Graph comparing 1.8-2-fold (solid bars) or 1.5-fold (patterned bars) gain of specific chromosomes within the population in the non-evolved (grey) and evolved (orange) bub3Δ lines. A binomial test was performed to determine whether the observed gain of the chromosomes in the CNV plot was statistically significant over any other chromosome. For the test, the expected proportion of successful events (aneuploidy) was 1.5 for non-evolved lines (grey dashed line) and 1.8 for evolved lines (orange dashed line). (C) Graph of the percent of bub3Δ lines with the listed number of extra copies of the chromosomes.

To our surprise, all *bub3*Δ lines that we sequenced were aneuploid, despite the minimal number of cell divisions prior to sequencing (Figs 1E and S3A). Of the 16 chromosomes in budding yeast, the gained chromosomes were restricted to chromosomes I, II, III, VIII, and X, with chromosome II present in 8 of the 11 lines (Fig 2A2B). The lines had between 1-4 extra chromosomes, with most having 2 extra chromosomes (Fig 2C). These results were unexpected for several reasons. First, we did not expect the rapid accumulation of aneuploidy in the lines, as aneuploidy generally causes a growth disadvantage. Second, while budding yeast can tolerate the aneuploidies of most chromosomes, the consistent accumulation of the same five chromosomes was unexpected [4]. Third, the aneuploid chromosomes varied in size, including both short and long chromosomes, not only the short chromosomes that may be more tolerable. Therefore, we hypothesized that the gained chromosomes may give *bub3*Δ cells an advantage to outcompete the euploid population.

### *The bub3*Δ lines have a dynamic karyotype over generations

**Fig 3:**
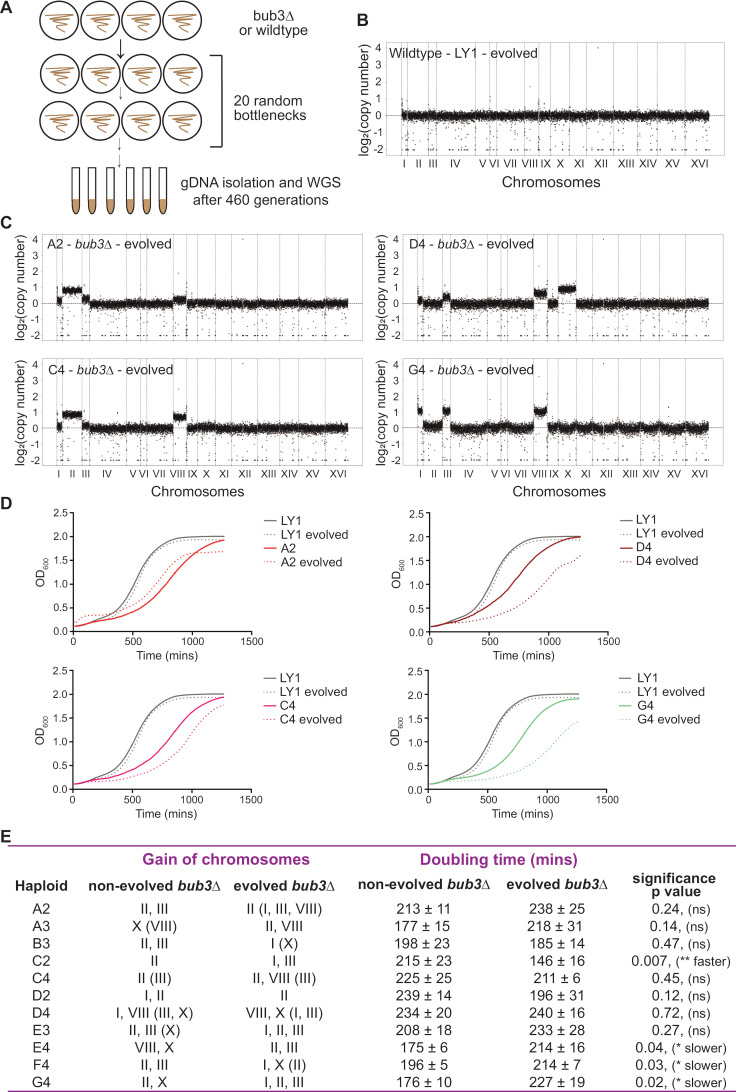
Evolved *bub3*Δ lines continue to gain one or more specific chromosomes and do not necessarily grow faster. **(A)** Workflow of the evolution of wildtype or *bub3*Δ lines for whole genome sequencing after 460 generations. **(B-C)** CNV plots of evolved wildtype (B) or *bub3*Δ (C) karyotypes from whole genome sequencing. The x-axis shows the 16 budding yeast chromosomes separated by vertical dotted lines according to their sizes. The y-axis shows the log_2_(copy number). The horizontal line signifies 1 chromosome copy, and each increment shows an additional chromosome copy. **(D)** Growth curves comparing evolved (dotted lines) and non-evolved (solid lines) *bub3*Δ lines to a wildtype evolved and non-evolved line (LY1; in grey). The cells were grown to saturation, diluted to 0.1 OD_600_ and then the OD_600_ was measured for 20 hours. **(E)** Table comparing the gain of chromosomes and doubling times for non-evolved and evolved *bub3*Δ lines (using non-linear regression to measure the change of OD_600_ from 0.5 to 1). The significance shows the p-value comparing the doubling time of the evolved *bub3*Δ line to the non-evolved *bub3*Δ line (3 replicates; Unpaired t-test with Welch’s correction).

We hypothesized that if the gained chromosomes were beneficial, then *bub3*Δ cells would retain those chromosomes. Therefore, we asked if the karyotypes of the aneuploid *bub3*Δ lines stabilized after several generations. To this end, we evolved the original wildtype or *bub3*Δ lines by putting them through 20 random bottlenecks, which corresponded to ~460 generations (Fig 3A). This evolution scheme does not select for better growth, we were simply asking if the chromosomes were retained throughout the evolution. The whole genome sequencing showed that the evolved cells acquired few mutations that were not shared among independently evolved lines (S1 Table). CNV analysis showed that the wildtype euploid lines maintained their euploid karyotypes, and the wildtype aneuploid lines became less aneuploid, as expected (Figs 3B and S2B). The evolved *bub3*Δ lines showed the persistence of one or more extra chromosomes, still restricted to chromosomes – I, II, III, VIII, and X (Figs 2A–C,3C and S3B). Strikingly, the karyotypes were dynamic, such that different chromosomes were lost or gained over generations. Evolved lines showed an increased prevalence of chromosome I and were more likely to have 3–4 extra chromosomes than the non-evolved lines (Fig 2A–C). Overall, these results suggest that although aneuploidy was retained, the karyotypes did not stabilize, instead, the cells are actively gaining and losing the same 5 chromosomes over generations.

We were surprised that a gain of a specific chromosome could be present throughout the population within each line. We performed a binomial test to determine whether the gain of a specific chromosome (instead of any other yeast chromosome) occurred in enough sequenced lines to be statistically significant. In the non-evolved *bub3*Δ lines, the presence of chromosome II, III, and X was significant. In the evolved *bub3*Δ lines, the maintenance of chromosome I, II, and III was significant (Fig 2B).

### The evolved *bub3*Δ lines show variable growth rates compared to the non-evolved *bub3*Δ lines

The loss of important regulatory genes affects the survival and growth of the cells. Previous studies have shown that cells with mutations in regulatory genes occasionally gain chromosomes or more mutations over time for better survival and growth [9,11–16]. Thus, we asked if the persistent gain of specific chromosomes by evolved *bub3*Δ lines might provide a growth advantage. To test this, we compared the growth of evolved vs non-evolved *bub3*Δ and wildtype lines. To analyze the growth, we diluted the overnight grown cells to OD_600_ of 0.1 and measured the OD_600_ readings for the next 20 hours. The doubling times confirm that the *bub3*Δ lines grow slower as compared to wildtype and most of the evolved *bub3*Δ lines have growth rates that are similar to the unevolved lines (7/11 lines; Figs 3D, 3E and S4A). Only 1 evolved line grows faster and 3 grow slower. A comparison of the CNV plots and growth curves of *bub3*Δ cells shows that there is neither an obvious correlation between the growth rate and the specific chromosomes gained, nor the number of extra chromosomes (Fig 3E). These results suggest that the cells show chromosomal instability, but still only maintain the same 5 restricted chromosomes (I, II, III, VIII, and X).

### Loss of Bub3 results in an equal probability of chromosome missegregation across all tested chromosomes, but only specific chromosomes are maintained in the population

The gain of these five specific chromosomes in *bub3*Δ lines could be due to either a selective gain of the chromosomes or due to the maintenance of chromosomes that were missegregated. To distinguish between these possibilities, we compared the segregation of chromosomes II, III, IV, V and VIII upon acute Bub3 depletion. We chose these chromosomes because II, III, and VIII have an increased copy number in *bub3*Δ lines and IV, and V do not. Additionally, these chromosomes encompass short (chromosome III), medium (chromosomes V and VIII) and long (chromosomes II and IV) chromosome lengths. If chromosome gain was selective, we would expect only chromosome II, III, and VIII to show an increased copy number in the first cell cycles after Bub3 depletion. In contrast, if there were an equal likelihood of aneuploidy but only specific chromosomes were maintained, we would expect that there would be an equal probability of aneuploidy of all of the chromosomes in the first cell cycles after Bub3 depletion.

**Fig 4:**
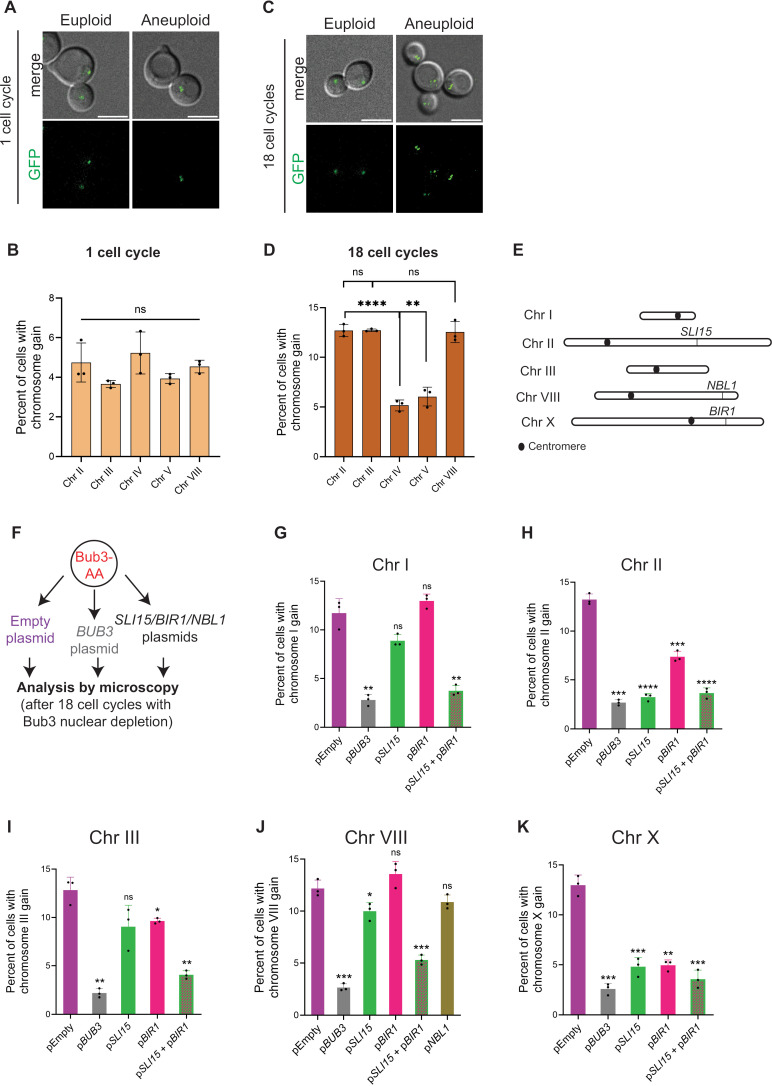
Increased expression of *SLI15* and *BIR1* prevents aneuploidy of specific chromosomes upon Bub3 depletion. **(A)** Representative images of euploid and aneuploid cells after the 1^st^ cell cycle of Bub3 depletion. Chromosome II was tagged with a LacO array in cells expressing LacI-GFP. Cells were released from an alpha-factor arrest with rapamycin addition to deplete Bub3 and imaged after the first cell cycle. Scale bar = 5µm. **(B)** Graph comparing the percent aneuploidy of chromosomes II, III, IV, V and VIII after the 1^st^ cell cycle of Bub3 depletion (n ≥ 500 cells per replicate; Unpaired t-test with Welch’s correction, error bars show standard deviation). (C) Representative images of euploid and aneuploid cells after 18 cell cycles of Bub3 depletion. Chromosome II is tagged with a LacO array in cells expressing LacI-GFP. Scale bar = 5µm. (D) Graph comparing the percent aneuploidy of chromosomes II, III, IV, V and VIII after 18 cell cycles of Bub3 depletion. **(E)** Schematic of the chromosomes gained in *bub3*Δ lines with genes of interest marked. The sizes are comparable by scale. **(F)** Workflow of the increased expression experiment. **(G-K)** The percent of cells with chromosome gain measured after 18 cell cycles of Bub3 depletion by counting corresponding chromosomes tagged with LacO in cells expressing LacI-GFP. Cells also have increased expression of CPC members *SLI15* (green), *BIR1* (pink), and *NBL1* (olive green) and with *SLI15* and *BIR1* combined (green with pink pattern) compared to strains with the control plasmids pEmpty (purple) and p*BUB3* (grey) comparing chromosomes I (G), II (H), III (I), VIII (J), and X (K) (n ≥500 cells each replicate; significance with Unpaired t-test with Welch’s correction; error bars represent standard deviation).

To monitor these chromosomes, we added a LacO array on the chromosomes in cells that expressed GFP-LacI, which tagged the chromosome with a GFP focus [34]. Cells with one focus in mother and daughter were scored as euploid (Fig 4A). In contrast, cells with extra GFP foci were scored as aneuploid. To monitor only one cell cycle in the absence of Bub3, we released the cells from G1 arrest and used the anchor away technique to deplete Bub3 from the nucleus upon rapamycin addition [28,29]. In this strain, Bub3 was tagged with FRB (FKBP12-rapamycin binding), and ribosomal protein Rpl13a was tagged with FKBP12 (FK binding protein 12)[35]. With rapamycin binding, FRB and FKBP12 stably interact, resulting in the removal of Bub3 from the nucleus as Rpl13a travels out of the nucleus. The strain also contains *fpr1*Δ and *tor1-1* to allow survival in the presence of rapamycin. We refer to this strain as Bub3-aa (Bub3 anchor away).

After synchronizing the cells with the α-factor, we added rapamycin and imaged cells after one cell cycle with Bub3 nuclear depletion. Approximately 4-5% of cells missegregated the tested chromosomes (Fig 4A and 4B). With Bub3 depletion for approximately 18 cell cycles, the percentage of cells with a gain of chromosomes II, III, and VIII were increased significantly compared to chromosomes IV and V, representing an accumulation of aneuploid cells within the population (Fig 4C and 4D). These results suggest that all chromosomes have an equal likelihood of chromosome missegregation, but only specific chromosomes are retained in the population. Overall, this result supports the model that selective increased copy number of specific chromosomes may provide an advantage to Bub3-depleted cells.

### Increased expression of *SLI15* and *BIR1* prevents aneuploidy of specific chromosomes when Bub3 is depleted from the nucleus

These results led us to hypothesize that increased expression of one or more genes on the aneuploid chromosomes may benefit *bub3*Δ cells, thereby contributing to the retention of these aneuploid chromosomes. If the increased copy number of a gene is advantageous to cells lacking Bub3, we would predict that increased expression of the gene on a plasmid would prevent the retention of the aneuploid chromosome upon Bub3 depletion.

To test this prediction, we started our analysis with three genes that encode components of the CPC and are present on the gained chromosomes: *SLI15* (chromosome II), *NBL1* (chromosome VIII), and *BIR1* (chromosome X) (Fig 4E). We cloned *SLI15, NBL1*, and *BIR1* with their endogenous promoters onto a centromere-containing (*CEN*) plasmid and transformed them into the Bub3-aa strains expressing LacI-GFP and with chromosomes of interest tagged with a LacO array (Fig 4F). As controls, we also transformed an empty plasmid and a *BUB3*-containing plasmid. We then assessed whether the elevated expression of those genes could prevent aneuploidy of chromosomes I, II, III, VIII, and X by counting GFP foci (Fig 4G-K).

Consistent with our prior results, approximately 10-15% of cells with the empty plasmid were aneuploid for each chromosome upon Bub3 depletion. The expression of *BUB3* reduced the aneuploidy to approximately 3% upon Bub3 depletion. Elevated expression of *SLI15* and *BIR1* reduced the aneuploidy of the chromosomes containing that specific gene, chromosome II and X to 2-5%, respectively (Fig 4H and 4K). In contrast, *NBL1* expression did not decrease the aneuploidy of chromosome VIII (Fig 4J). Interestingly, *SLI15* expression also reduced aneuploidy of chromosome X and modestly VIII (Fig 4J-K). *BIR1* expression also reduced aneuploidy of chromosome II and modestly III (Fig 4H-I). We therefore tested double expression of *SLI15* and *BIR1* and found that the aneuploidy of all 5 chromosomes was reduced (Fig 4G-K). We also find that increased expression of *SLI15* or *BIR1* allows cells to grow somewhat better upon Bub3 depletion, as shown through spot assays and growth curves (S5 Fig). Overall, these results suggest that increased expression of CPC components *SLI15* and *BIR1* benefit cells that lack Bub3.

### Increased expression of several specific genes on chromosome III prevents aneuploidy of chromosome III when Bub3 is depleted

**Fig 5:**
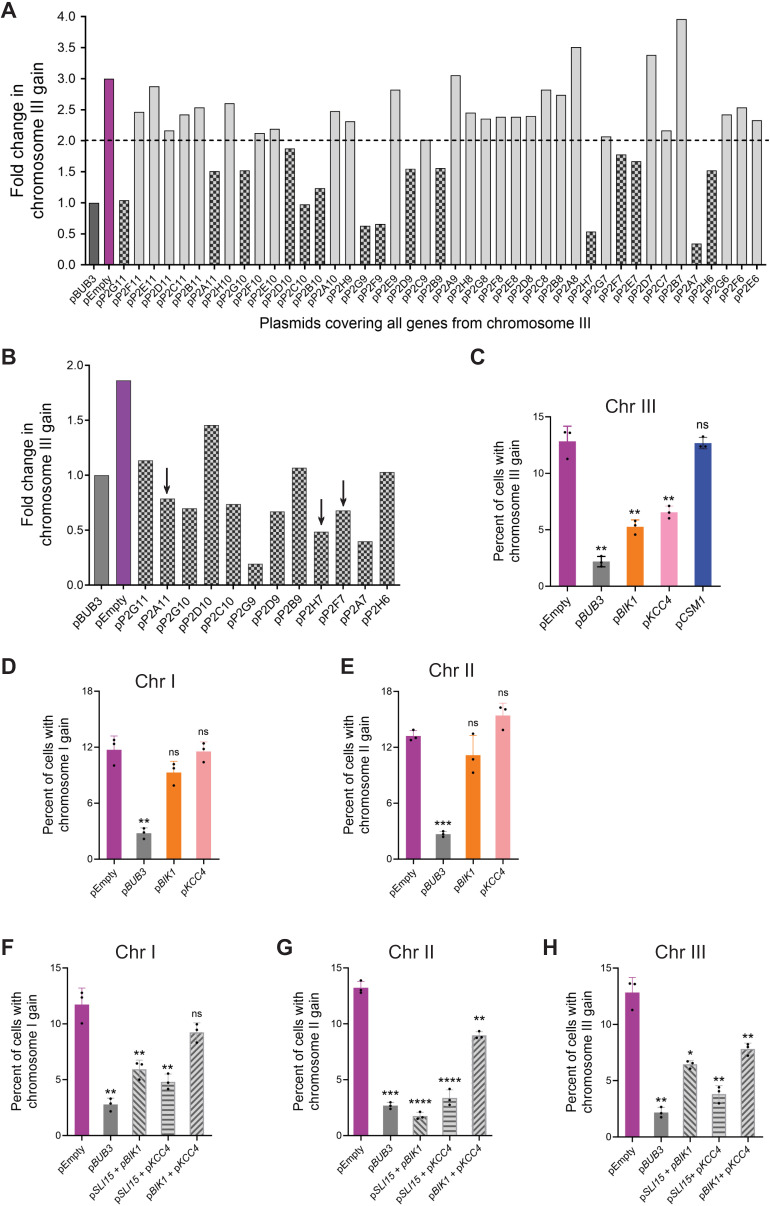
Elevated expression of a subset of genes from chromosome III prevents the gain of chromosome III upon Bub3 depletion. **(A-H)** Chromosome gain was measured by tagging the corresponding chromosome with LacO in cells expressing LacI-GFP. GFP foci were counted after Bub3 depletion for 18 cell cycles. **(A)** Graph comparing the fold-change in chromosome III gain for Bub3-aa strains with the plasmids of interest from the Yeast Tiling plasmid collection that spans chromosome III, p*BUB3* (dark grey) and pEmpty (purple) (n ≥ 500 cells counted per strain). The dotted line shows the cut-off of short-listed candidate plasmids. Plasmids under the line were chosen for secondary screening (patterned bars). **(B)** The graphs showing the secondary screen of the short-listed candidates. Plasmids with the genes of interest are marked with an arrow. **(C**-H) Graph of the percent aneuploidy after 18 cell cycles of Bub3 depletion and with increased expression of the genes of interest: (C) *BIK1*, *KCC4*, and *CSM1* for chromosome III; (D, E) *BIK1* and *KCC4* for chromosome I and II; (F-H) double expression of the genes of interest for chromosomes I, II, III (n ≥ 500 cells for each replicate; significance with Unpaired t-test with Welch’s correction; error bars represent SD).

Chromosomes I, III, and VIII did not have any obvious candidates for genes that could benefit *bub3*Δ cells upon chromosome gain. Because chromosome III is the most highly represented aneuploidy after chromosome II in both the non-evolved and evolved *bub3*Δ strains, we decided to screen for genes on chromosome III that prevent aneuploidy upon Bub3 depletion (Fig 2A and 2B). We hypothesized that if increased expression of a specific gene provided a benefit to *bub3*Δ cells, the presence of that gene on a plasmid could prevent the aneuploidy of chromosome III because it would not need to maintain the aneuploidy. We isolated the 2µ plasmids from the Yeast Tiling Collection that spanned chromosome III, individually transformed them in Bub3-aa strains expressing LacI-GFP and with a LacO array on chromosome III, and then monitored chromosome III segregation after 18 cell cycles with Bub3 nuclear depletion by monitoring the GFP foci [36]. From this analysis, we found 15 plasmids that could decrease the likelihood of chromosome III gain below 2-fold (there was a 3-fold increased likelihood of chromosome III gain in cells with the empty plasmid as compared to cells with the *BUB3* plasmid; Fig 5A). Of the 15 plasmids, 3 had overlapping genes. Therefore, we re-tested 12 plasmids that reduced chromosome III gain upon Bub3 nuclear depletion (Fig 5B).

We were surprised that so many plasmids reduced chromosome III aneuploidy upon Bub3-depletion. This result suggests that multiple genes likely provide a benefit to cells that lack Bub3. The 2µ plasmids in the Yeast Tiling Collection contain between 4 to 11 genes in each plasmid (S2 Table)[36]. To narrow down the list, we focused on candidates with known roles in chromosome segregation or cell cycle regulation - *BIK1*, *KCC4,* and *CSM1*. We subcloned those genes in *CEN* plasmids and transformed them into Bub3-aa strains. *CEN* plasmids are present in lower copy numbers than the 2µ plasmids, allowing a similar comparison to increasing the copy number through chromosome gain by aneuploidy. After nuclear depletion of Bub3 for 18 cell cycles, cells expressing *CSM1* did not reduce the percent of cells with chromosome III aneuploidy, suggesting that a different gene on the Tiling plasmid likely reduces the chromosome III aneuploidy (Fig 5C). In contrast, elevated expression of *BIK1* and *KCC4* had less aneuploidy of chromosome III than cells with the empty plasmid (Fig 5C). Bik1 is a microtubule-associated protein that is important for chromosome segregation and spindle elongation [31,37–40]. Kcc4 is involved in the G2/M checkpoint [41–43]. Therefore, these genes likely benefit the Bub3-depleted cells by enhancing chromosome segregation.

We next asked if the expression of *BIK1* and *KCC4* could prevent the aneuploidy of other chromosomes. We monitored chromosomes I and II and found no significant difference in the percent of aneuploidy upon Bub3 depletion (Fig 5D and 5E). However, double expression of both *BIK1* and *KCC4* could prevent gain of chromosomes II and III, but not I (Fig 5F-H). We note that expression of both *BIK1* and *KCC4* does not reduce the percent of chromosome III gain to levels as low as expression of *BUB3,* suggesting that other genes on that chromosome may also provide a benefit. The double expression of *BIK1* and *SLI15* or *KCC4* and *SLI15* prevented aneuploidy of chromosomes I, II, and III (Fig 5F-H). This was interesting because the single expression of *SLI15* did not reduce aneuploidy of chromosomes I or III (Fig 4G and 4I). These results suggest that there could be synergistic effects from increased expression of specific genes on different chromosomes.

Finally, we checked whether increased expression of *BIK1* and *KCC4* allowed the Bub3-depleted cells to grow faster. The spot assay and growth curve (S5 Fig) showed that the *BIK1* and *KCC4* individually or together only modestly improves cell growth. Overall, we conclude that by increasing the copy number of the chromosome through aneuploidy, the increased expression of several genes provides *bub3*Δ cells a benefit, likely by reducing chromosome instability, but not necessarily by improving cell growth.

### The evolved *bub3*Δ cells maintain aneuploidy after reintroduction of *BUB3*

**Fig 6:**
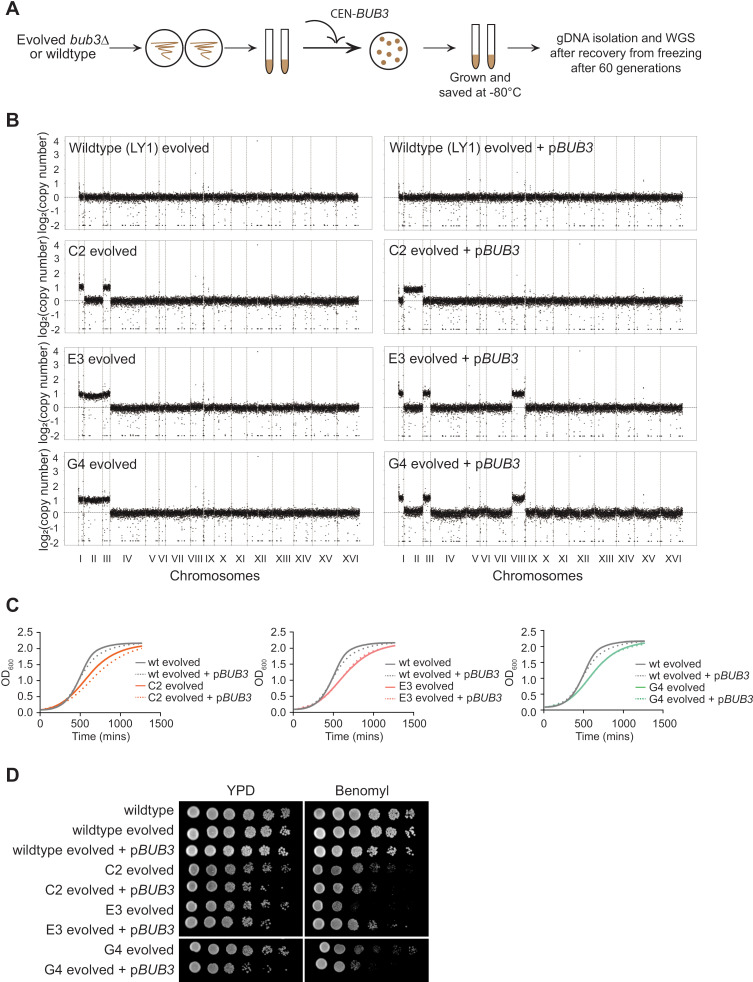
The re-introduction of *BUB3* in evolved *bub3*Δ lines does not rescue aneuploidy. **(A)** Workflow for *BUB3* reintroduction in evolved *bub3*Δ lines for whole genome sequencing. **(B)** CNV plots comparing the evolved *bub3*Δ lines with and without the rescue plasmid from whole genome sequencing. The x-axis shows the 16 budding yeast chromosomes separated by vertical dotted lines according to their sizes. The y-axis shows the log_2_(copy number). The horizontal line signifies 1 chromosome copy, and each increment shows an additional chromosome copy. **(C)** Growth curve comparing the evolved *bub3*Δ lines and a wildtype line before and after addition of the rescue plasmid. **(D)** 1:10 serial dilutions of saturated cultures comparing the benomyl sensitivity of evolved *bub3*Δ lines with and without p*BUB3* (5μg/mL of benomyl).

We wondered if the evolved aneuploid *bub3*Δ lines could recover a euploid genotype by adding *BUB3* back to the cells. We tested three evolved *bub3*Δ lines, C2, E3, and G4, and transformed them with a *CEN* plasmid containing *BUB3* (Fig 6A). We then froze them down and struck them out again and grew them for whole genome sequencing. We purposefully treated them the same as our original assay that identified the *bub3*Δ aneuploid lines after a minimal number of generations, thinking that they would be able to lose the aneuploid chromosomes over the approximately 60 generations if they were no longer providing a benefit to the cells. Surprisingly, the sequencing revealed that all three lines were aneuploid and had different aneuploid karyotypes from the starting evolved lines (Fig 6B). Although we expected that growth would improve after *BUB3* addition, growth assays showed that strain C2 grew slower after *BUB3* addition, but the other strains showed similar growth with and without *BUB3* addition (Fig 6C). On a low concentration of the microtubule-depolymerizing drug benomyl, only strain E3 showed better growth upon *BUB3* addition (Fig 6D). Overall, these results suggest that the evolved *bub3*Δ cells have a chromosome instability phenotype that was not overcome quickly after adding *BUB3* back.

## Discussion

Our study reveals that *bub3*Δ haploid lines rapidly gain 5 specific chromosomes in budding yeast: I, II, III, VIII, and X (Fig 2). Most of the lines gained at least 2 chromosomes, with chromosome II highly represented. These chromosomes are a variety of sizes with both short and long chromosomes represented, suggesting that they did not preferentially maintain only the shorter chromosomes. Our results suggest that all chromosomes have an equal probability of chromosome missegregation upon Bub3 depletion, but that only specific chromosomes were retained (Fig 4 A-D). These results led us to hypothesize that the extra copies of these chromosomes may provide a benefit to *bub3*Δ cells, likely due to increased expression of specific genes on those chromosomes.

Previous studies have also reported that the gain of specific chromosomes occurs in cells that have mutations of different regulatory genes [11–16]. A relevant example analyzed the rare *bir1*Δ survivors [11,15]. Bir1 is an essential component of the CPC, but approximately 10% of *bir1*Δ spores can grow into a colony. When these survivors were further evolved for better growth, and sequenced, the evolved strains had an increased growth rate, and the same 5 chromosomes were gained as found in our study. A notable difference in our study is that we sequenced the *bub3*Δ strains both before and after evolving them and found the same 5 chromosomes gained before evolving them. Furthermore, we did not select for better growth with the evolution; we were simply asking if the chromosomes were retained through 460 generations. However, the evolved strains had more complex karyotypes with an increase in the number of chromosomes retained (Fig 2). Most of the evolved strains do not grow faster than the non-evolved strains, so the advantage provided to the cells is likely through decreasing the amount of aneuploidy, not by providing faster growth. Our results suggest that the karyotype, which represents a snapshot at the time of whole genome sequencing, may provide a benefit to the cells by reducing the overall probability of chromosome missegregation.

We found the similarity of the gain of the same 5 chromosomes in both *bub3*Δ cells and *bir1*Δ cells surprising because loss of *BIR1* has a much more severe phenotype. Yet, both proteins are involved in recruiting Ipl1/Aurora B to the inner centromere [27]. The *bub3*Δ cells have a less severe phenotype than *bir1*Δ cells because multiple redundant pathways bring the CPC to the kinetochore and the loss of Bub3 only disrupts one of them [27–29]. Furthermore, the evolved *bir1*Δ lines also had additional mutations that were not found in the *bub3*Δ lines, suggesting that these mutations were likely to further help with the survival of *bir1*Δ cells [11,15]. Combined, these results suggest that there are specific genes whose increased copy number provides a mutual benefit to cells with an increased probability of chromosome missegregation.

In both *bub3*Δ and *bir1*Δ cells, the increased copy number of *SLI15* acquired through the gain of chromosome II provided a benefit to the cells by reducing aneuploidy of other chromosomes [11](Fig 4). Furthermore, increasing the copy number of CPC component *BIR1* can also provide a benefit to Bub3-depleted cells (Fig 4). The increased expression of *SLI15* and *BIR1* singly prevents aneuploidy of chromosomes II and X upon Bub3 depletion. Increased expression of both prevents aneuploidy of all 5 chromosomes upon Bub3 depletion. Furthermore, a previous study showed that *bub1*Δ cells cannot survive as tetraploids, but their viability is rescued with increased expression of *BIR1* and *SLI15* [44]. These results suggest that the increased copy number of these two CPC components may provide a benefit by decreasing the probability of chromosome missegregation.

There were no obvious candidates on the other three chromosomes that could potentially provide a benefit to *bub3*Δ cells with increased expression. We therefore screened all the genes on chromosome III and surprisingly identified 12 plasmids from the Yeast Tiling Collection that reproducibly prevented aneuploidy (Fig 5). We focused only on two candidates: the microtubule-binding protein *BIK1* and a G2/M checkpoint regulator *KCC4* [38–43]. The increased expression of *BIK1* and *KCC4* reduced aneuploidy of chromosome III upon Bub3 depletion, but not of chromosome I or II (Fig 5C-E). The increased expression of both *BIK1* and *KCC4* reduced aneuploidy of the other chromosomes to different extents (Fig 5F-H). However, the increased expression of these genes provides only a modest growth advantage (S5 Fig). These results suggest that the increased expression of both *BIK1* and *KCC4* provides an additive benefit to Bub3-depleted cells to decrease chromosome missegregation, but not necessarily by improving growth.

Interestingly, our screen showed that there are likely many other genes, in addition to *BIK1* and *KCC4,* that prevent aneuploidy of chromosome III (Fig 5A and 5B). There were no other obvious candidates involved in chromosome segregation, but these genes may be involved in other processes that provide an advantage to Bub3-depleted cells and may be interesting for further study. Similarly, besides Nbl1, which did not prevent aneuploidy upon Bub3 depletion, there were no obvious candidates on chromosomes I and VIII. However, our combined results suggest that many genes on chromosomes I, II, III, VIII, and X are likely to provide a benefit to *bub3*Δ cells when gained through aneuploidy, giving an advantage to the cells that retain those chromosomes. We note that this benefit is likely through the reduction in chromosome missegregation, not through increased growth rates. Therefore, the additive benefits of multiple genes may allow strains with lower chromosome segregation fidelity to survive.

## Materials and methods

### Yeast and plasmid strains

All *S. cerevisiae* strains are derived from the W303 strain background and are listed in S3 Table. All gene deletions, gene tagging, and self-replicating plasmid introductions were performed using the standard PCR-based lithium acetate transformation method [45]. The plasmids to fluorescently tag genes (LacI-GFP and Tub1-mRuby) were integrated into the genome [34,46]. The wildtype or *bub3*Δ haploids used for the evolution were obtained by dissecting tetrads from a *BUB3*/*bub3*Δ diploid strain (LY4387) to avoid initial aneuploidy. The anchor-away strains have *tor1-1* mutation and *fpr1*Δ to avoid rapamycin toxicity [35]. *RPL13A* was tagged with 2xFKBP12 and *BUB3* was tagged with FRB to allow their interaction in the presence of rapamycin to deplete Bub3 from the nucleus.

All plasmids and primers used in this study are listed in S4 and S5 Tables, respectively. The *CEN* plasmids were cloned using restriction digestion by PCR amplifying the genes of interest with their endogenous promoters from genomic DNA or a Tiling plasmid. The primers had flanking restriction enzyme sites for the cloning (mentioned in S5 Table). The PCR products were subcloned in *CEN* plasmids (S4 Table).

### Media and growth conditions

All yeast strains were grown at 30°C. All yeast strains except the ones transformed with a *CEN* or 2µ plasmid were grown in media containing 1% yeast extract, 2% peptone, and 2% glucose (YPD). The yeast strains transformed with the Yeast Tiling plasmid collection were grown in YPD supplemented with G418. *CEN* plasmid-containing yeast strains were grown in synthetic dropout media containing 0.67% yeast nitrogen base without amino acids, 2% glucose (SC), and 0.2% dropout amino acid mix. The Yeast Tiling plasmid collection plasmids (2µ plasmids) were isolated from bacteria grown on LB plates (or media) supplemented with 50µg/mL of kanamycin [36]. The other bacterial plasmids were grown on LB plates (or media) supplemented with 100µg/mL of ampicillin. The plasmids were isolated using QIAprep Spin Miniprep kit.

### Evolution of wildtype or mutant *BUB3* haploids

To minimize aneuploidy, we used a wildtype diploid and then deleted one copy of *BUB3* to get a heterozygous *BUB3*/*bub3*::LEU2 diploid (LY4387). We sporulated the diploids and then dissected the tetrads. The 4-spore viable tetrads were grown up in YPD and frozen. They were then restreaked and grown up in YPD for genomic DNA preparation for sequencing. To obtain the evolved wildtype or *bub3*Δ cells, the non-evolved strains underwent 20 random bottlenecks in which the plates were marked prior to streaking and colonies closest to the mark were restreaked for the next bottleneck, allowing a random selection of the colonies. The 20 passages account for approximately 460 generations. The evolved strains were grown up for freezing and then restreaked and grown up for genomic DNA preparation for sequencing.

### Whole Genome Sequencing analysis

#### Library preparation.

Input DNA was quantified by Qubit (Thermo Fisher) and 200ng was used as input into the Nextera DNA with tagmentation workflow (Illumina) to generate Illumina sequencing libraries according to the manufacturer’s protocol. Libraries were normalized and pooled for sequencing on a NextSeq2000 (Illumina), targeting 10 million, 150 bp paired-end reads per sample.

#### Read Alignment.

Raw reads were trimmed with Trimmomatic v0.39 [47], with the following parameters, “ILLUMINACLIP:TruSeq3-PE-2.fa:2:30:10:2:keepBothReads LEADING:3 TRAILING:3 MINLEN:36”. Trimmed reads were aligned to the Yeast genome S288C, available at NCBI accession GCF_000146045.2, using BWA 0.7.17 [48], with default parameters. Duplicate reads were marked with “MarkDuplicates” from Picard tools v2.27.1, with default parameters. The raw data were uploaded to Geo at https://www.ncbi.nlm.nih.gov/bioproject/PRJNA1194573.data

#### SNP and Indel Analysis.

SNPs and small indels were called with FreeBayes v1.3.4 [49] and the following parameters “--min-coverage 5 --limit-coverage 200 --min-alternate-fraction.2 --min-mapping-quality 15 --min-alternate-count 2”. SNPs were annotated using SNPEff v5.0e [50].

#### Copy Number Analysis.

Copy number analysis was performed using the following commands from the Genome Analysis Tool Kit (GATK) v4.5.0.0 [51]. Genome intervals for calculating copy number were determined with the PreprocessIntervals command, with the following parameters, “--padding 0 -imr OVERLAPPING_ONLY”. Reads counts for each sample and each interval were collected using the CollectReadCounts command, with default parameters. Copy number per interval was standardized and denoised using the DenoiseReadCounts command, with the “--standardized-copy-ratios” and “--denoised-copy-ratios” parameters. Genome-wide copy number graphs were created by plotting columns 2 (“START”) and 4 (“LOG2_COPY_RATIO”) of the denoised copy ratio output.

### Spot assays

Strains were grown in YPD for 18–20hrs at 30°C. The saturated cultures were serially diluted 1:10 and spotted on YPD plates and incubated at 30°C for 40 hours.

### Growth curve analysis

Strains were grown in YPD for 18–20hrs at 30°C. The cultures were diluted to 0.1 OD_600_ in YPD. OD_600_ readings were taken with the Synergy Neo2 plate reader every 10 minutes in triplicates for approximately 20 hours.

### Increased expression screen for chromosome III genes

The Yeast Tiling Collection plasmids were isolated and 96-well plate transformations were performed as follows [36,52]. LY9391 was grown in 20mL of YPD for 12 hours at 30°C. 2.5 x 10^8^ cells were transferred to 50ml of pre-warmed YPD and incubated at 30°C for 4 hours. The culture was spun down and the pellet was resuspended in 15mL of media. 200µL of cells were transferred to 96-well plates and the plates were centrifuged for 10 minutes at 1300g. The supernatant was discarded. 5µL of the Yeast Genomic Tiling Collection plasmids were added to each well. 35µL of the transformation mix (15µL of 1M lithium acetate + 20µL of boiled 2mg/mL single-stranded salmon sperm DNA) and 100µL of 50% PEG (MW 3350) were added to the cell pellet and incubated at 42°C for 2 hours. The plate was centrifuged at 1300g for 10 minutes. The supernatant was discarded and 10µL of sterile water was added to the cells and 5µL of cells were spotted on SC-leu plates. The plates were incubated at 30°C for 24 hours and then replica-plated on YPD+G418 plates for 2–4 days at 30°C. The single colonies obtained from the transformation were restreaked on YPD+G418 and then grown up and frozen down. For the increased expression assay, cells were recovered from the frozen stocks, and then grown on YPD+G418 plates with or without rapamycin (1µg/ml) for 24 hours at 30°C. A random single colony was incubated in YPD+G418 media with or without rapamycin (1µg/mL) for 6 hours at 30°C. The culture was spun down and washed with SC media. 500 cells were scored in each sample as either euploid or aneuploid using LacO-LacI-GFP foci.

### Increased expression screen for individual genes

To analyze the effect of increased expression of CPC genes and chromosome III candidates obtained from the increased expression screen, the genes of interest were cloned in a *CEN*-plasmid as listed in S4 Table and transformed in yeast strains containing LacO arrays on the chromosomes of interest as listed in S3 Table. The frozen stocks of the transformed yeast strains were recovered on appropriate SC dropout plates and a random fully grown colony was streaked on plates with and without Rapamycin (1µg/mL) for 24 hours at 30°C. A random single colony was incubated in appropriate SC dropout media with or without Rapamycin (1µg/mL) for 6 hours at 30°C. 500 cells were scored for each sample as either euploid or aneuploid using LacO-LacI-GFP foci.

### Statistical analysis

The statistical analysis for all graphs was done using GraphPad Prism 10.2.2. The two-tailed P values were calculated using the Unpaired t-test with Welch’s correction. The significance is as follows: **** < 0.0001, *** < 0.001, ** < 0.01, ns > 0.05.

A binomial test was performed using GraphPad Prism determine if the gain of the specific chromosomes observed in the CNV plots was statistically significant (Fig 2B). For the test, the expected proportion of successful events (aneuploidy) was calculated as follows:

Expected proportion = total number of aneuploid events/ total number of chromosomes. This expected value represents the likelihood of aneuploidy being treated as a successful event in the analysis. For the non-evolved population, our expected value was 1.5 and for the evolved population, our expected number was 1.8.

## Supporting information

S1 Fig Spindle checkpoint mutants differentially affect cellular growth and morphology. **(A)** Growth curves comparing wildtype and individual spindle checkpoint deletion strains. **(B)** The doubling time of wildtype and spindle checkpoint deletion strains, measured by non-linear regression for a change in OD_600_ from 0.5 to 1 (3 replicates from two individual experiments; Unpaired t-test with Welch’s correction; error bars represent standard deviation). **(C)** Representative DIC images comparing morphological differences of wildtype and spindle checkpoint mutant strains (scale bar = 5µm).
(PDF)

S2 Fig Wildtype lines maintain normal chromosome copy numbers. **(A, B)** The CNV plots of wildtype non-evolved (A) and evolved (B) lines from whole genome sequencing. The x-axis shows the 16 yeast chromosomes spaced by vertical dotted lines according to their sizes. The horizontal dotted line shows 1 chromosome copy. Each increment shows an additional chromosome copy.
(PDF)

S3 Fig Bub3Δ lines have varying chromosome copy numbers over time. **(A, B)** The CNV of *bub3*Δ non-evolved (A) and evolved (B) lines from whole genome sequencing. The x-axis shows the 16 budding yeast chromosomes spaced by vertical dotted lines according to their sizes. The horizontal dotted line shows 1 chromosome copy. Each increment shows an additional chromosome copy.
(PDF)

S4 Fig The evolution of *bub3Δ* lines does not provide a growth advantage in most lines. **(A)** Growth curves comparing evolved (dotted lines) to non-evolved (solid lines) wildtype and *bub3*Δ lines.
(PDF)

S5 Fig The increased expression of one or more advantageous genes modestly improves growth. (A) Comparison of the Bub3-aa strains with control plasmids (pEmpty or p*BUB3*) and single or double *CEN* plasmids. Saturated yeast cultures were serially diluted, spotted on YPD plates with and without Rapamycin to a final concentration of 1μg/mL, and imaged after 40 hours of incubation. The comparison of doubling times (by measuring the non-linear regression in the change of OD_600_ from 0.5 to 1). The cells were grown to saturation, diluted to 0.1 OD_600_ and the OD_600_ was measured for 20 hours.
(PDF)

S1 Table SNP analysis. List of all single nucleotide polymorphisms (SNPs) identified in the bub3Δ lines. (PDF)

S2 Table Gene candidates from chromosome III secondary screen. List of the genes present on each plasmid from the Yeast Tiling Plasmids that were rescreened in Fig 5B.
(PDF)

S3 Table Yeast strains. List of yeast strains used in this study. All strains were from the W303 background and have the following mutations: *ade2-1 his3-11,15 leu2-3,112 trp1-1 ura3-1* and *can1-100.*
(PDF)

S4 Table Plasmid list. List of plasmids used in this study. (PDF)

S5 Table Primer list. List of primers used in this study. (PDF)

S6 Table Reagent list. Reagents used in this study. (PDF)

S1 Data SNP data. Excel file of the sequencing data from SNP analysis found in S1 Table.
(XLSX)

S2 Data Microscopy data. Excel file of the raw data from the microscopy experiments.
(XLSX)
