# Supplementary figures and images for: Aneuploidy of specific chromosomes is beneficial to cells lacking spindle checkpoint protein Bub3
Source: PLoS Genet. 2025 Feb 4;21(2):e1011576. doi: 10.1371/journal.pgen.1011576 (PMC11819610; doi:10.1371/journal.pgen.1011576)

Supplementary figure 1

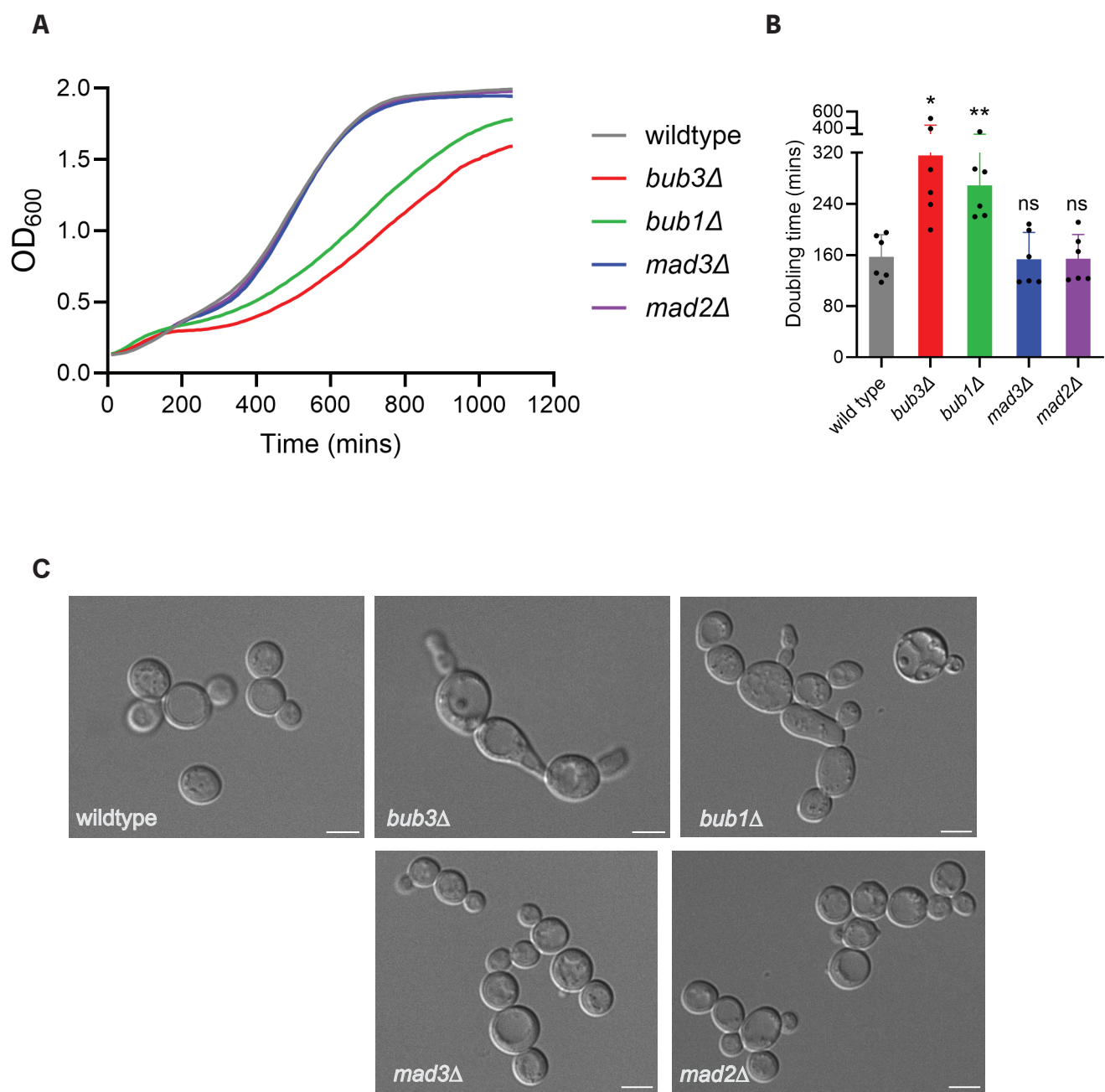

Supplement: S1 Fig — (A) Growth curves comparing wildtype and individual spindle checkpoint deletion strains. (B) The doubling time of wildtype and spindle checkpoint deletion strains, measured by non-linear regression for a change in OD600 from 0.5 to 1 (3 replicates from two individual experiments; Unpaired t-test with Welch’s correction; error bars represent standard deviation). (C) Representative DIC images comparing morphological differences of wildtype and spindle checkpoint mutant strains (scale bar = 5µm). (PDF) [file pgen.1011576.s001.pdf]

Supplementary Figure 2

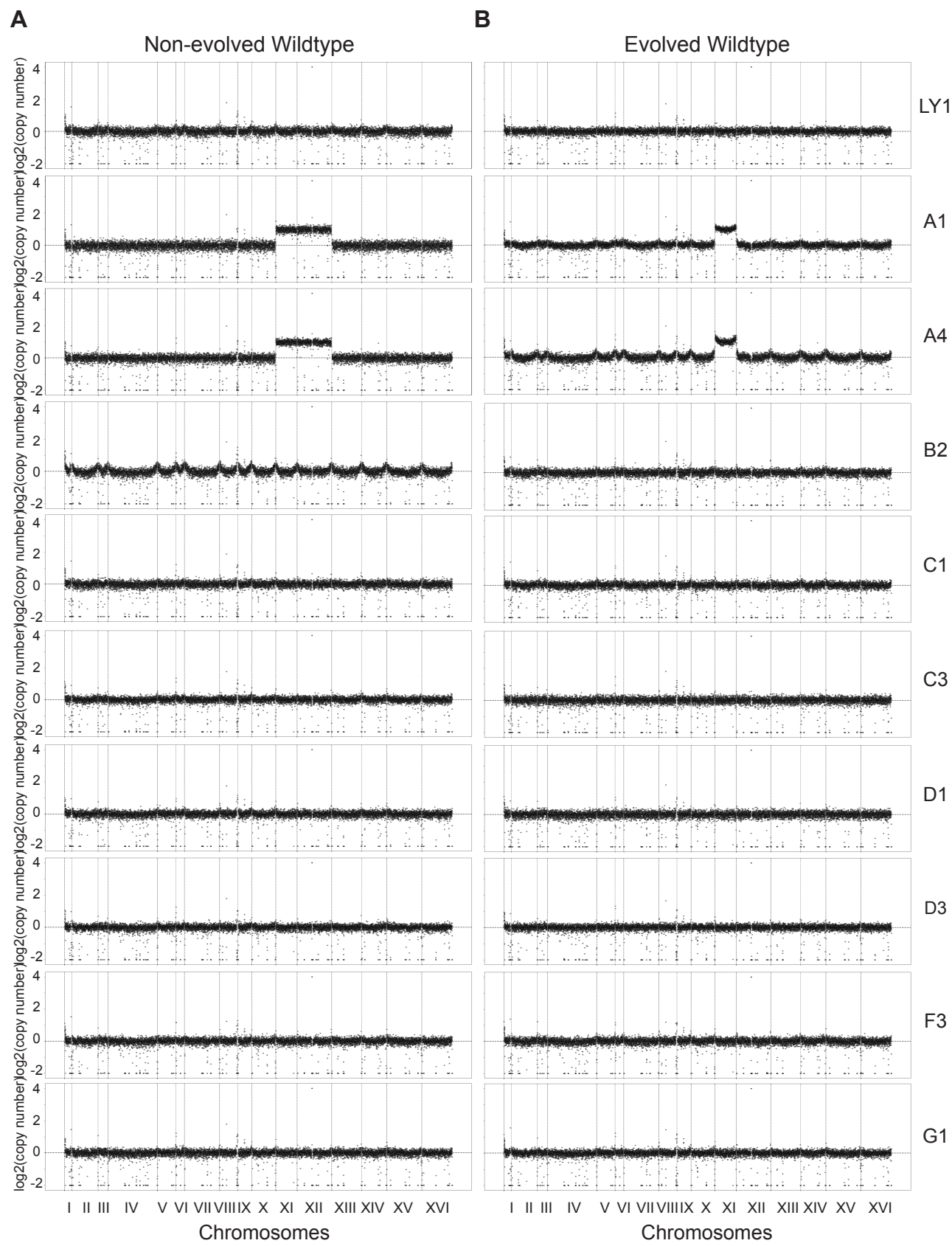

Supplement: S2 Fig — (A, B) The CNV plots of wildtype non-evolved (A) and evolved (B) lines from whole genome sequencing. The x-axis shows the 16 yeast chromosomes spaced by vertical dotted lines according to their sizes. The horizontal dotted line shows 1 chromosome copy. Each increment shows an additional chromosome copy. (PDF) [file pgen.1011576.s002.pdf]

Supplementary Figure 3

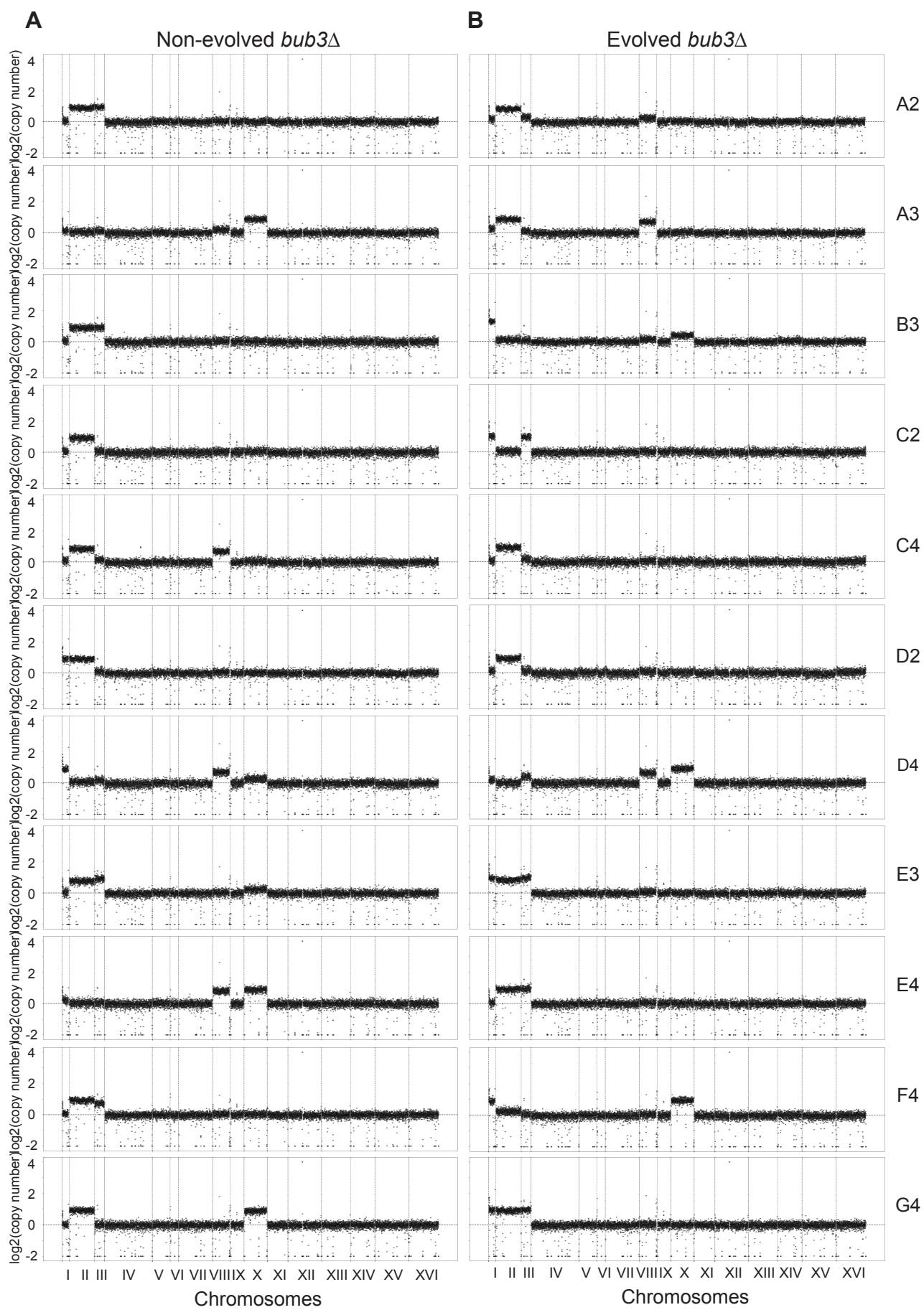

Supplement: S3 Fig — (A, B) The CNV of bub3Δ non-evolved (A) and evolved (B) lines from whole genome sequencing. The x-axis shows the 16 budding yeast chromosomes spaced by vertical dotted lines according to their sizes. The horizontal dotted line shows 1 chromosome copy. Each increment shows an additional chromosome copy. (PDF) [file pgen.1011576.s003.pdf]

Supplementary figure 4

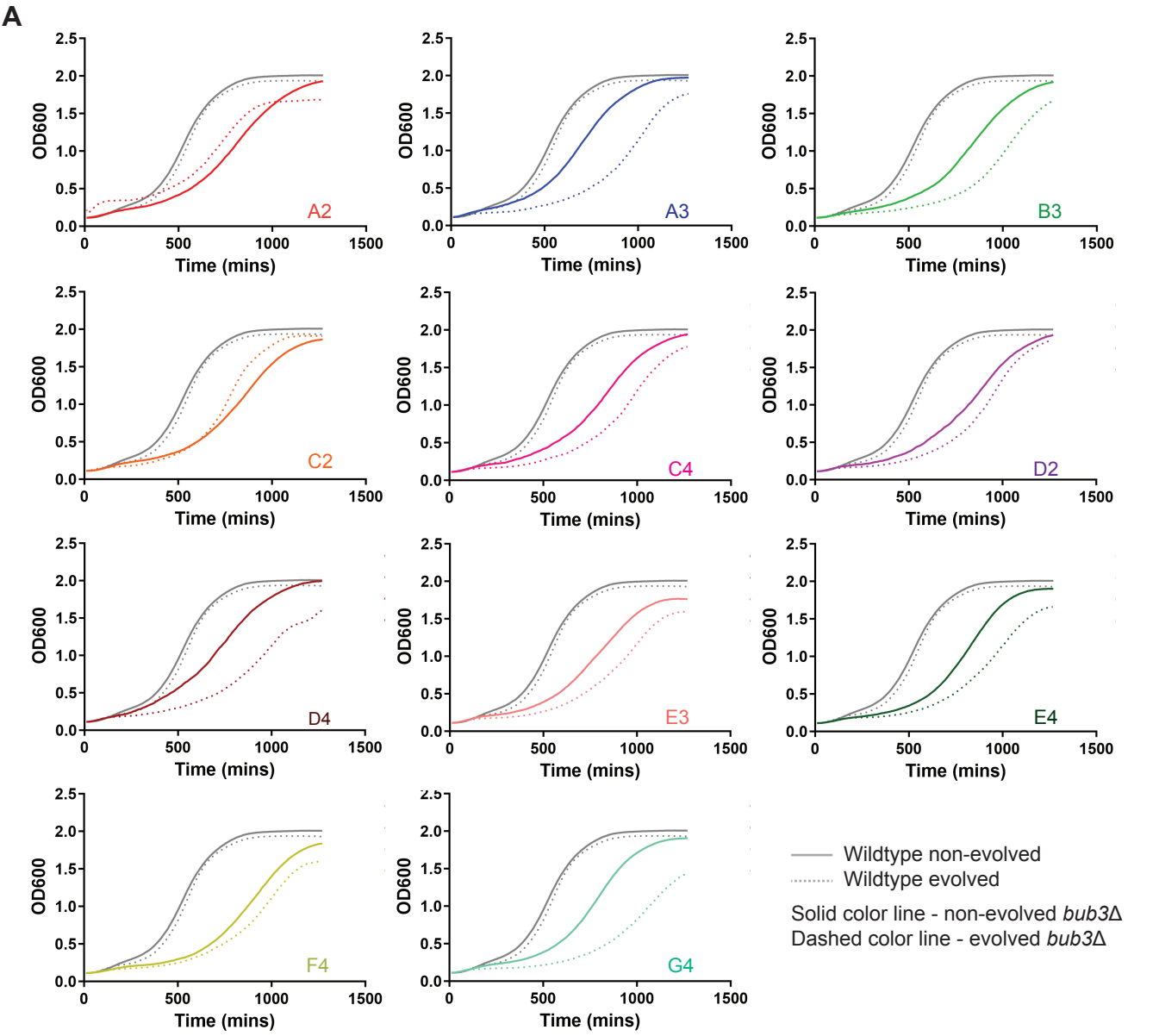

Supplement: S4 Fig — (A) Growth curves comparing evolved (dotted lines) to non-evolved (solid lines) wildtype and bub3Δ lines. (PDF) [file pgen.1011576.s004.pdf]

Supplementary Fig 5

A

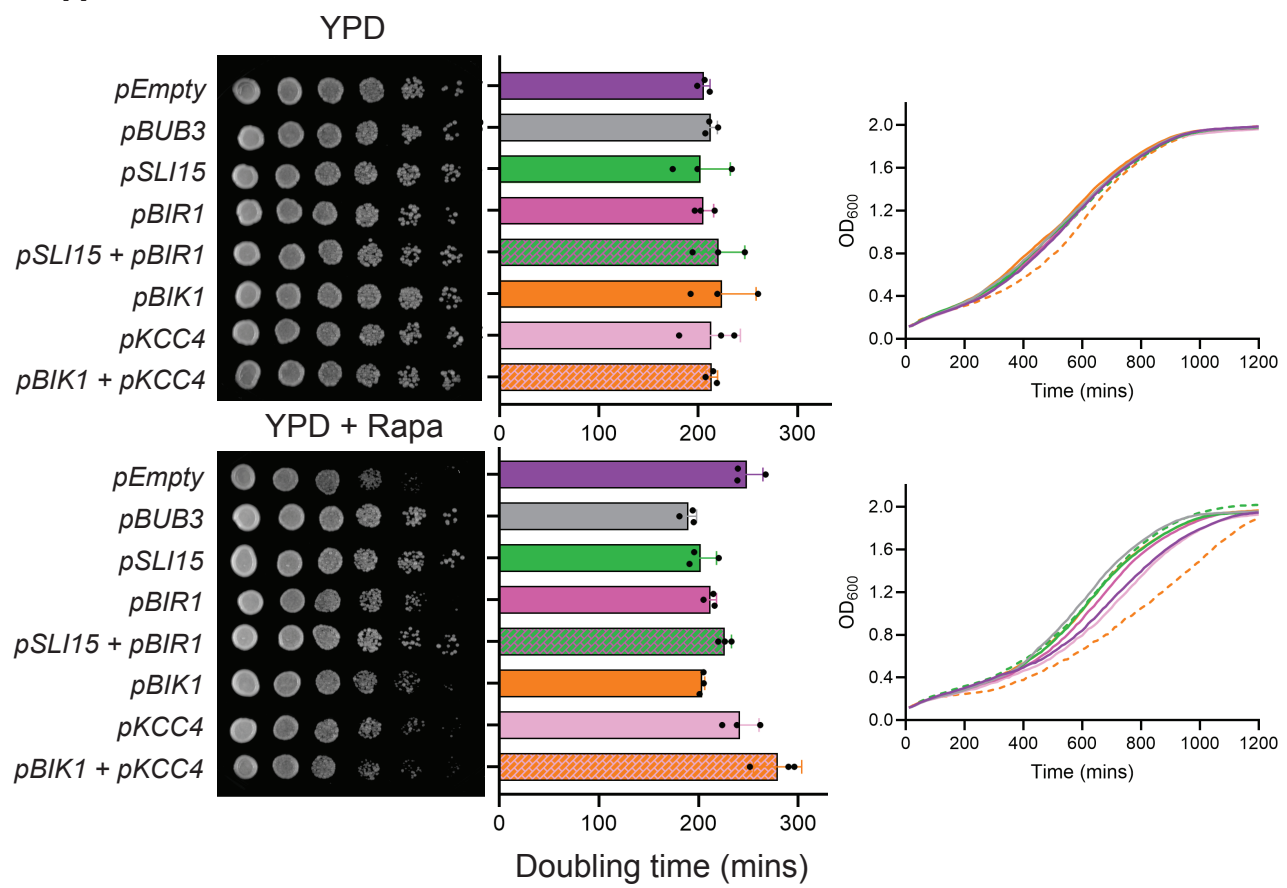

Supplement: S5 Fig — (A) Comparison of the Bub3-aa strains with control plasmids (pEmpty or pBUB3) and single or double CEN plasmids. Saturated yeast cultures were serially diluted, spotted on YPD plates with and without Rapamycin to a final concentration of 1μg/mL, and imaged after 40 hours of incubation. The comparison of doubling times (by measuring the non-linear regression in the change of OD600 from 0.5 to 1). The cells were grown to saturation, diluted to 0.1 OD600 and the OD600 was measured for 20 hours. (PDF) [file pgen.1011576.s005.pdf]
