# Supplementary material for: Aneuploidy of specific chromosomes is beneficial to cells lacking spindle checkpoint protein Bub3
Source: PLoS Genet. 2025 Feb 4;21(2):e1011576. doi: 10.1371/journal.pgen.1011576 (PMC11819610; doi:10.1371/journal.pgen.1011576)
Supplement: S1 Table — (PDF) [file pgen.1011576.s006.pdf]

S1_Table: SNP analysis

All *bub3*Δ haploids (non-evolved and evolved) have the following mutations in *LEU2,* which is introduced while deleting *BUB3.*

**Nucleotide change**	**Amino acid change**	**Mutation type**
248dupG	Thr84fs	frameshift_variant
791dupG	Leu265fs	frameshift_variant

SNPs identified in *bub3*Δ haploids -

	**Strain**		**Gene**	**Nucleotide change**	**Amino acid change**	**Mutation type**
**1**	LY10897	evolved	YMR317W	c.786A>G	Ser262Ser	synonymous_variant
**2**	LY10900	non-evolved	YIR042C	c.3625A>G		upstream_gene_variant
**3**	LY10901	evolved	YIR042C	c.3625A>G		upstream_gene_variant
			tW(UCA)Q	c.-2388T>G		upstream_gene_variant
			ATP8	c.-2170A>T		upstream_gene_variant
			ATP8	c.-2165_-2164insTTATAA		upstream_gene_variant
			ATP6	c.529_531delTTCinsATT		missense_variant
			ATP6	c.714_723delCATTCAGGGAinsTATCCAATCT		missense_variant
			ATP6	c.729_738delCTGGGCTATTinsTTGACTTATC		stop_gained
			COX1	c.*2665_*2670delTATCTAinsCATATC		downstream_gene_variant
			COX1	c.*2675_*2676insT		downstream_gene_variant
			COX1	c.*2680T>C		downstream_gene_variant

			COX1	c.*2709_*2712delCAAGinsAAAA		downstream_gene_variant
			COX1	c.*2736delCinsTT		downstream_gene_variant
			COX1	c.*2757_*2758insT		downstream_gene_variant
			COX1	c.*2768C>T		downstream_gene_variant
			COX1	c.*2788C>T		downstream_gene_variant
			COX1	c.*2870delT		downstream_gene_variant
			COX1	c.*2893A>T		downstream_gene_variant
			COX1	c.*2921C>T		downstream_gene_variant
			COX1	c.*2927A>G		downstream_gene_variant
			COX1	c.*3262_*3263insA		downstream_gene_variant
			COX1	c.*3444_*3445insTTA		downstream_gene_variant
			COX1	c.*3516C>A		downstream_gene_variant
			COX1	c.*3557C>A		downstream_gene_variant
			tE(UUC)Q	c.-4388C>A		upstream_gene_variant
			tE(UUC)Q	c.-4215T>C		upstream_gene_variant
			tE(UUC)Q	c.-1906T>C		upstream_gene_variant

			tE(UUC)Q	c.-1899_-1898insG		upstream_gene_variant
			tE(UUC)Q	c.-1862_-1843delAATATAATATAATATAATAT		upstream_gene_variant
			tE(UUC)Q	c.-1290_-1285delTAAATA		upstream_gene_variant
			tE(UUC)Q	c.-1276_-1275insT		upstream_gene_variant
			tE(UUC)Q	c.-966G>T		upstream_gene_variant
			tE(UUC)Q	c.-925_-924insTATTTAATATTTAATATTTAATATTTAATATTTAA		upstream_gene_variant
			tE(UUC)Q	c.-896C>A		upstream_gene_variant
			tE(UUC)Q	c.-755_-754insAATAAT		upstream_gene_variant
			tE(UUC)Q	c.-685_-682delTAAT		upstream_gene_variant
			tE(UUC)Q	c.-641_-638delAAAT		upstream_gene_variant
			BI2	c.-528G>T		upstream_gene_variant
			OLI1	c.-3631_-3630insT		upstream_gene_variant
			VAR1	c.508_510delAAT		conservative_inframe_deletion
			VAR1	c.600A>T		missense_variant
			OLI1	c.*4040delT		downstream_gene_variant
			OLI1	c.*4623delTinsAGTTCCGGGCCCCGGCCACGGGAGCCGGAACCCCGGAAGGA		downstream_gene_variant
			21S_RRNA	n.-3433_-3432insG		upstream_gene_variant

			21S_RRNA	n.-2515_-2514insTTTTATTTAATTTTATTTAATTTTATTTAA		upstream_gene_variant
			21S_RRNA	n.-2264T>A		upstream_gene_variant
			21S_RRNA	n.-1838G>A		upstream_gene_variant
			21S_RRNA	n.-1821G>T		upstream_gene_variant
			21S_RRNA	n.-1779T>C		upstream_gene_variant
			21S_RRNA	n.-1742_-1721delGGTCCGCCCCCGCGTGGGCGGA		upstream_gene_variant
			21S_RRNA	n.-1048A>T		upstream_gene_variant
			21S_RRNA	n.-1005A>T		upstream_gene_variant
			21S_RRNA	n.-972C>T		upstream_gene_variant
			21S_RRNA	n.-936T>A		upstream_gene_variant
			21S_RRNA	n.-871delT		upstream_gene_variant
			21S_RRNA	n.-849G>T		upstream_gene_variant
			21S_RRNA	n.-835delAinsTTAT		upstream_gene_variant
			21S_RRNA	n.-806_-792delATTTCTCCTTTCTTAinsGGAACTTA		upstream_gene_variant
			21S_RRNA	n.-745_-741delCTCTT		upstream_gene_variant
			21S_RRNA	n.-728_-726delCCAinsTTC		upstream_gene_variant
			SCEI	c.-2471A>T		upstream_gene_variant
			SCEI	c.-1809dupT		upstream_gene_variant

**4**	LY10909	evolved	TIF3	c.110C>A	p.Thr37Lys	missense_variant
**5**	LY10910	non-evolved	GEM1	c.1359C>T	p.Val453Val	synonymous_variant
**6**	LY10911	evolved	TAF2	c.2338A>G	p.Arg780Gly	missense_variant
			BOI2	c.58G>A	p.Asp20Asn	missense_variant
**7**	LY10912	non-evolved	STR2	c.1748C>A	pThr583Lys	missense_variant
			RPL38	c.-3155_-3154insA		upstream_gene_variant
**8**	LY10913	evolved	STR2	c.1748C>A	pThr583Lys	missense_variant
			RPL38	c.-3155_-3154insA		upstream_gene_variant
**9**	LY10921	evolved + *pBUB3*	DSF2	c.-4042T>C		upstream_gene_variant
			TAF2	c.2338A>G	p.Arg780Gly	missense_variant
			BOI2	c.58G>A	p.Asp20Asn	missense_variant
**10**	LY10914	non-evolved	YLL066W-B	c.96_98delCCAinsACCACACC	p.His33fs	frameshift_variant and missense_variant
			YLL066C	c.-1129A>G		
**11**	LY10915	evolved	YLL066W-B	c.96_98delCCAinsACCACACC	p.His33fs	frameshift_variant and missense_variant
			YLL066C	c.-1129A>G		upstream_gene_variant
			YLL067C	c.-1553_-1550delCATGinsAATA		upstream_gene_variant
**12**	LY10916	non-evolved	PAU8	c.-1520A>T		upstream_gene_variant
			PAU8	c.-1501C>G		upstream_gene_variant
			PAU8	c.-1457C>T		upstream_gene_variant
			PAU8	c.-1448G>A		upstream_gene_variant

			PAU8	c.-1410C>G		upstream_gene_variant
			PAU8	c.-1346delA		upstream_gene_variant
			PAU8	c.-1330G>T		upstream_gene_variant
			PAU8	c.-1321_-1311delTCACTCCATGGinsCCACA		upstream_gene_variant
			PAU8	c.-1297G>A		upstream_gene_variant
			PAU8	c.-1196G>A		upstream_gene_variant
**13**	LY10917	evolved	YLL066W-B	c.96_98delCCAinsACCACACC	p.His33fs	frameshift_variant and missense_variant
			YLL066C	c.-1129A>G		
**14**	LY10922	evolved + *pBUB3*	YLL066W-B	c.96_98delCCAinsACCACACC	p.His33fs	frameshift_variant and missense_variant
			YLL066C	c.-1129A>G		
