# Supplementary material for: Aneuploidy of specific chromosomes is beneficial to cells lacking spindle checkpoint protein Bub3
Source: PLoS Genet. 2025 Feb 4;21(2):e1011576. doi: 10.1371/journal.pgen.1011576 (PMC11819610; doi:10.1371/journal.pgen.1011576)
Supplement: S2 Table — List of the genes present on each plasmid from the Yeast Tiling Plasmids that were rescreened in Fig 5B. (PDF) [file pgen.1011576.s007.pdf]

S2_Table: Gene candidates from chromosome III secondary screen

Plasmid	Gene	Gene name
P2G11	ADH7	Alcohol dehydrogenase
	RDS1	Regulator of drug sensitivity
	AAD3	Aryl-alcohol dehydrogenase
	[YCR108C]	
P2A11	[SRB8]&	Suppressor of RNA Polymerase B
	YCR081C-A	
	AHC2	Ada histone acetyltransferase complex component
	TRX3	Thioredoxin
	TUP1	dTMP-Uptake
	YCR085W	
	CSM1	Chromosome segregation in meiosis
	YCR087W	
	YCR087C-A	
	ABP1	Actin binding protein
	[FIG2]*	Factor-induced gene
P2G10	[SSK22]*	Suppressor of sensor kinase
	SOL2	Suppressor of Los1-1
	ERS1	ERD suppressor
	EGO2	Exit from Rapamycin induced growth arrest
	FUB1	Function of Boundary
	[PAT1]	Protein associated with Topoisomerase II
	[YCR079W]*	Phosphatase Two C
P2D10	[BPH1]*	Beige Protein homolog
	[PWP2]*	Periodic tryptophan (W) protein
	YIH1	Yeast Impact Homolog
	TAH1	Tpr-containing protein associated with Hsp90
	TVS1	Transmembrane protein vital for stress response
	tS(CGA)C	
	YCR064C	
	BUD31	Bud site selection
	HCM1	High copy suppressor of calmodulin
	RAD18	Radiation sensitive
	[SED4]&	Suppressor of Erd2 deletion
P2C10	[THR4]	Threonine requiring
	CTR86	Copper transport protein
	PWP2	Periodic tryptophan (W) protein
	[YIH1]	Yeast Impact Homolog
	[TAH1]*	Tpr-containing protein associated with Hsp90
P2G9	[BPH1]&	Beige Protein homolog

	SNT1<br>FEN1<br>RRP43<br>RBK1<br>[PHO87]&	Sant domain<br>Fatty acid elongation<br>Ribosomal RNA processing<br>Ribokinase<br>Phosphate metabolism
P2D9	FEN2<br>RIM1<br>SYP1<br>snR65<br>RPS14A<br>snR189	Fenprompimorph resistance<br>Replication in mitochondria<br>Suppressor of yeast profilin deletion<br>small nucleolar RNA<br>Ribosomal protein of small subunit<br>small nucleolar RNA
P2H7	[AGP1]*<br>YCL023C<br>KCC4<br>YCL022C<br>tE(UUC)C<br>YCL021W-A<br>YCL019W<br>YCL020W	High-affinity glutamine permease
P2F7	[RRP7]*<br>HIS4<br>BIK1<br>RNQ1<br>FUS1<br>HBN1<br>FRM2<br>[AGP1]&	Ribosomal RNA processing<br>Histidine requiring<br>Bilateral karyogamy effect<br>Rich in Asparagine and Glutamine<br>cell fusion<br>Homologous to bacterial nitroreductase<br>Fatty acid repression mutant<br>High-affinity glutamine permease
P2A7	[SPB1]&<br>PBN1<br>LRE1<br>APA1<br>[YCL049C]	Suppressor of PaB1 mutant<br>Protease B Non-derepressible<br>Laminarase resistance<br>AP4A phosphorylase
P2H6	[KRR1]*<br>FYV5<br>[YCL058W-A]<br>MIC10<br>PRD1<br>PEX34<br>KAR4<br>SPB1<br>[PBN1]	contains KRR-R motif<br>Function required for yeast viability<br>Antisense of Depressing Factor<br>Mitochondrial contact site and cristae formation<br>proteinase yscD<br>peroxin<br>Karyogamy<br>Suppressor of PaB1 mutant<br>Protease B Non-derepressible
