# Supplementary material for: Aneuploidy of specific chromosomes is beneficial to cells lacking spindle checkpoint protein Bub3
Source: PLoS Genet. 2025 Feb 4;21(2):e1011576. doi: 10.1371/journal.pgen.1011576 (PMC11819610; doi:10.1371/journal.pgen.1011576)
Supplement: S3 Table — All strains were from the W303 background and have the following mutations: ade2-1 his3-11,15 leu2-3,112 trp1-1 ura3-1 and can1-100. (PDF) [file pgen.1011576.s008.pdf]

S3_Table: Yeast strains

Strain number	Strain genotype
LY1	Mat a
LY2	Mat α
LY427	Mat a, Tub1-GFP:URA3, Spc42-mCherry:KanMX, *mad2*::LEU2
LY483	Mat a/α
LY1717	Mat a, Tub1-GFP:URA3, Spc42-mCherry:KanMX, *bub1*::KanMX
LY1940	Mat a, Tub1-GFP:URA3, Spc42-mCherry:KanMX, *bub3*::LEU2
LY1949	Mat a, Tub1-GFP:URA3, Spc42-mCherry:KanMX
LY2098	Mat a, Tub1-GFP:URA3, Spc42-mCherry:KanMX, *mad3*::KanMX
LY4387	Mat a/α, *bub3*::LEU2/*BUB3*
LY9021	Mat a, *tor1-1*, *fpr1*::NatMX, RPL13A-2xFKBP12:loxP, Bub3-FRB:KanMX, Tub1-mRuby2:URA3, CUP1prLacI-GFP:HIS3, LacO:LEU2 (Chr III)
LY9391	Mat a, *tor1-1*, *fpr1*::NatMX, RPL13A-2xFKBP12:loxP, Bub3-FRB:HphMX, Tub1-mRuby2:URA3, CUP1prLacI-GFP:HIS3, LacO:LEU2 (Chr III)
LY9513	Mat a, *tor1-1*, *fpr1*::NatMX, RPL13A-2xFKBP12:loxP, Bub3-FRB:HphMX, Tub1-mRuby2:URA3, CUP1prLacI-GFP:HIS3, LacO:LEU2 (Chr III), *BUB3*-2μ:LEU2:KanMX
LY9531	Mat a, *tor1-1*, *fpr1*::NatMX, RPL13A-2xFKBP12:loxP, Bub3-FRB:HphMX, Tub1-mRuby2:URA3, CUP1prLacI-GFP:HIS3, LacO:LEU2 (Chr III), 2μ:LEU2:KanMX
LY10346	Mat a, *tor1-1*, *fpr1*::NatMX, RPL13A-2xFKBP12:loxP, Bub3-FRB:KanMX, Tub1-mRuby2:HphMX, CUP1prLacI-GFP:HIS3, LacO:TRP1 (Chr II)
LY10348	Mat a, *tor1-1*, *fpr1*::NatMX, RPL13A-2xFKBP12:loxP, Bub3-FRB:KanMX, Tub1-mRuby2:HphMX, CUP1prLacI-GFP:HIS3, LacO:TRP1 (Chr IV)
LY10349	Mat a, *tor1-1*, *fpr1*::NatMX, RPL13A-2xFKBP12:loxP, Bub3-FRB:KanMX, Tub1-mRuby2:HphMX, CUP1prLacI-GFP:HIS3, LacO:LEU2 (Chr V)
LY10437	Mat a, *tor1-1*, *fpr1*::NatMX, RPL13A-2xFKBP12:loxP, Bub3-FRB:KanMX, Tub1-mRuby2:HphMX, CUP1prLacI-GFP:HIS3, LacO:TRP1 (Chr II), CEN:URA3
LY10438	Mat a, *tor1-1*, *fpr1*::NatMX, RPL13A-2xFKBP12:loxP, Bub3-FRB:KanMX, Tub1-mRuby2:HphMX, CUP1prLacI-GFP:HIS3, LacO:TRP1 (Chr II), *BUB3*-CEN:URA3
LY10439	Mat a, *tor1-1*, *fpr1*::NatMX, RPL13A-2xFKBP12:loxP, Bub3-FRB:KanMX, Tub1-mRuby2:HphMX, CUP1prLacI-GFP:HIS3, LacO:TRP1 (Chr II), *SLI15*-CEN:URA3
LY10440	Mat a, *tor1-1*, *fpr1*::NatMX, RPL13A-2xFKBP12:loxP, Bub3-FRB:KanMX, Tub1-mRuby2:HphMX, CUP1prLacI-GFP:HIS3, LacO:TRP1 (Chr II), *BIK1*-CEN:URA3
LY10449	Mat a, *tor1-1*, *fpr1*::NatMX, RPL13A-2xFKBP12:loxP, Bub3-FRB:KanMX, Tub1-mRuby2:HphMX, CUP1prLacI-GFP:HIS3, LacO:LEU2 (Chr III), CEN:URA3

LY10450	Mat a, *tor1-1*, *fpr1*::NatMX, RPL13A-2xFKBP12:loxP, Bub3-FRB:KanMX, Tub1-mRuby2:HphMX, CUP1prLacI-GFP:HIS3, LacO:LEU2 (Chr III), *BUB3*-CEN:URA3
LY10451	Mat a, *tor1-1*, *fpr1*::NatMX, RPL13A-2xFKBP12:loxP, Bub3-FRB:KanMX, Tub1-mRuby2:HphMX, CUP1prLacI-GFP:HIS3, LacO:LEU2 (Chr III), *BIK1*-CEN:URA3
LY10452	Mat a, *tor1-1*, *fpr1*::NatMX, RPL13A-2xFKBP12:loxP, Bub3-FRB:KanMX, Tub1-mRuby2:HphMX, CUP1prLacI-GFP:HIS3, LacO:LEU2 (Chr III), *SLI15*-CEN:URA3
LY10456	Mat a, *tor1-1*, *fpr1*::NatMX, RPL13A-2xFKBP12:loxP, Bub3-FRB:KanMX, Tub1-mRuby2:HphMX, CUP1prLacI-GFP:HIS3, LacO:TRP1 (Chr VIII)
LY10457	Mat a, *tor1-1*, *fpr1*::NatMX, RPL13A-2xFKBP12:loxP, Bub3-FRB:KanMX, Tub1-mRuby2:HphMX, CUP1prLacI-GFP:HIS3, LacO:TRP1 (Chr VIII), CEN:URA3
LY10458	Mat a, *tor1-1*, *fpr1*::NatMX, RPL13A-2xFKBP12:loxP, Bub3-FRB:KanMX, Tub1-mRuby2:HphMX, CUP1prLacI-GFP:HIS3, LacO:TRP1 (Chr VIII), *BUB3*-CEN:URA3
LY10460	Mat a, *tor1-1*, *fpr1*::NatMX, RPL13A-2xFKBP12:loxP, Bub3-FRB:KanMX, Tub1-mRuby2:HphMX, CUP1prLacI-GFP:HIS3, LacO:TRP1 (Chr VIII), *SLI15*-CEN:URA3
LY10461	Mat a, *tor1-1*, *fpr1*::NatMX, RPL13A-2xFKBP12:loxP, Bub3-FRB:KanMX, Tub1-mRuby2:HphMX, CUP1prLacI-GFP:HIS3, LacO:TRP1 (Chr VIII), *NBL1*-CEN:URA3
LY10468	Mat a, *tor1-1*, *fpr1*::NatMX, RPL13A-2xFKBP12:loxP, Bub3-FRB:KanMX, Tub1-mRuby2:HphMX, CUP1prLacI-GFP:HIS3, LacO:TRP1 (Chr X), CEN:URA3
LY10469	Mat a, *tor1-1*, *fpr1*::NatMX, RPL13A-2xFKBP12:loxP, Bub3-FRB:KanMX, Tub1-mRuby2:HphMX, CUP1prLacI-GFP:HIS3, LacO:TRP1 (Chr X), *BUB3*-CEN:URA3
LY10470	Mat a, *tor1-1*, *fpr1*::NatMX, RPL13A-2xFKBP12:loxP, Bub3-FRB:KanMX, Tub1-mRuby2:HphMX, CUP1prLacI-GFP:HIS3, LacO:TRP1 (Chr X), *SLI15*-CEN:URA3
LY10538	Mat a, *tor1-1*, *fpr1*::NatMX, RPL13A-2xFKBP12:loxP, Bub3-FRB:KanMX, Tub1-mRuby2:HphMX, CUP1prLacI-GFP:HIS3, LacO:LEU2 (Chr I), CEN:URA3
LY10539	Mat a, *tor1-1*, *fpr1*::NatMX, RPL13A-2xFKBP12:loxP, Bub3-FRB:KanMX, Tub1-mRuby2:HphMX, CUP1prLacI-GFP:HIS3, LacO:LEU2 (Chr I), *BUB3*-CEN:URA3
LY10540	Mat a, *tor1-1*, *fpr1*::NatMX, RPL13A-2xFKBP12:loxP, Bub3-FRB:KanMX, Tub1-mRuby2:HphMX, CUP1prLacI-GFP:HIS3, LacO:LEU2 (Chr I), *BIK1*-CEN:URA3
LY10541	Mat a, *tor1-1*, *fpr1*::NatMX, RPL13A-2xFKBP12:loxP, Bub3-FRB:KanMX, Tub1-mRuby2:HphMX, CUP1prLacI-GFP:HIS3, LacO:LEU2 (Chr I), *SLI15*-CEN:URA3

LY10627	Mat a, *tor1-1*, *fpr1*::NatMX, RPL13A-2xFKBP12:loxP, Bub3-FRB:KanMX, Tub1-mRuby2:HphMX, CUP1prLacI-GFP:HIS3, LacO:LEU2 (Chr III), *BIK1*-CEN:URA3, *SLI15*-CEN:TRP1
Ly10628	Mat a, *tor1-1*, *fpr1*::NatMX, RPL13A-2xFKBP12:loxP, Bub3-FRB:KanMX, Tub1-mRuby2:HphMX, CUP1prLacI-GFP:HIS3, LacO:LEU2 (Chr I), *BIK1*-CEN:URA3, *SLI15*-CEN:TRP1
LY10629	Mat a, *tor1-1*, *fpr1*::NatMX, RPL13A-2xFKBP12:loxP, Bub3-FRB:KanMX, Tub1-mRuby2:HphMX, CUP1prLacI-GFP:HIS3, LacO:TRP1 (Chr II), *SLI15*-CEN:URA3, *BIK1*-CEN:LEU2
LY10656	Mat a, *tor1-1*, *fpr1*::NatMX, RPL13A-2xFKBP12:loxP, Bub3-FRB:KanMX, Tub1-mRuby2:HphMX, CUP1prLacI-GFP:HIS3, LacO:TRP1 (Chr II), *CSM1*-CEN:URA3
LY10658	Mat a, *tor1-1*, *fpr1*::NatMX, RPL13A-2xFKBP12:loxP, Bub3-FRB:KanMX, Tub1-mRuby2:HphMX, CUP1prLacI-GFP:HIS3, LacO:LEU2 (Chr III), *CSM1*-CEN:URA3
LY10660	Mat a, *tor1-1*, *fpr1*::NatMX, RPL13A-2xFKBP12:loxP, Bub3-FRB:KanMX, Tub1-mRuby2:HphMX, CUP1prLacI-GFP:HIS3, LacO:LEU2 (Chr I), *CSM1*-CEN:URA3
LY10670	Mat a, *tor1-1*, *fpr1*::NatMX, RPL13A-2xFKBP12:loxP, CEN:URA3
LY10671	Mat a, *tor1-1*, *fpr1*::NatMX, RPL13A-2xFKBP12:loxP, *BUB3*-CEN:URA3
LY10744	Mat a, *tor1-1*, *fpr1*::NatMX, RPL13A-2xFKBP12:loxP, Bub3-FRB:KanMX, Tub1-mRuby2:HphMX, CUP1prLacI-GFP:HIS3, LacO:LEU2 (Chr III), *KCC4*-CEN:URA3
LY10746	Mat a, *tor1-1*, *fpr1*::NatMX, RPL13A-2xFKBP12:loxP, Bub3-FRB:KanMX, Tub1-mRuby2:HphMX, CUP1prLacI-GFP:HIS3, LacO:LEU2 (Chr I), *KCC4*-CEN:URA3
LY10746	Mat a, *tor1-1*, *fpr1*::NatMX, RPL13A-2xFKBP12:loxP, Bub3-FRB:KanMX, Tub1-mRuby2:HphMX, CUP1prLacI-GFP:HIS3, LacO:TRP1 (Chr II), *KCC4*-CEN:URA3
LY10759	Mat a, *tor1-1*, *fpr1*::NatMX, RPL13A-2xFKBP12:loxP, Bub3-FRB:KanMX, Tub1-mRuby2:HphMX, CUP1prLacI-GFP:HIS3, LacO:TRP1 (Chr II), *KCC4*-CEN:URA3, *BIK1*-CEN:LEU2
LY10760	Mat a, *tor1-1*, *fpr1*::NatMX, RPL13A-2xFKBP12:loxP, Bub3-FRB:KanMX, Tub1-mRuby2:HphMX, CUP1prLacI-GFP:HIS3, LacO:LEU2 (Chr III), *KCC4*-CEN:URA3, *BIK1*-CEN:TRP1
LY10761	Mat a, *tor1-1*, *fpr1*::NatMX, RPL13A-2xFKBP12:loxP, Bub3-FRB:KanMX, Tub1-mRuby2:HphMX, CUP1prLacI-GFP:HIS3, LacO:LEU2 (Chr I), *KCC4*-CEN:URA3, *BIK1*-CEN:TRP1
LY10762	Mat a, *tor1-1*, *fpr1*::NatMX, RPL13A-2xFKBP12:loxP, Bub3-FRB:KanMX, Tub1-mRuby2:HphMX, CUP1prLacI-GFP:HIS3, LacO:LEU2 (Chr III), *KCC4*-CEN:URA3, *SLI15*-CEN:TRP1
LY10763	Mat a, *tor1-1*, *fpr1*::NatMX, RPL13A-2xFKBP12:loxP, Bub3-FRB:KanMX, Tub1-mRuby2:HphMX, CUP1prLacI-GFP:HIS3, LacO:LEU2 (Chr I), *KCC4*-CEN:URA3, *SLI15*-CEN:TRP1

LY10766	Mat a, *tor1-1*, *fpr1*::NatMX, RPL13A-2xFKBP12:loxP, Bub3-FRB:KanMX, Tub1-mRuby2:HphMX, CUP1prLacI-GFP:HIS3, LacO:TRP1 (Chr II), *KCC4*-CEN:URA3, *SLI15*-CEN:LEU2
LY10802	Mat a, *tor1-1*, *fpr1*::NatMX, RPL13A-2xFKBP12:loxP, Bub3-FRB:KanMX, Tub1-mRuby2:HphMX, CUP1prLacI-GFP:HIS3, LacO:TRP1 (Chr II), *BIR1*-CEN:URA3
LY10814	Mat a, *tor1-1*, *fpr1*::NatMX, RPL13A-2xFKBP12:loxP, Bub3-FRB:KanMX, Tub1-mRuby2:HphMX, CUP1prLacI-GFP:HIS3, LacO:LEU2 (Chr III), *BIR1*-CEN:URA3
LY10815	Mat a, *tor1-1*, *fpr1*::NatMX, RPL13A-2xFKBP12:loxP, Bub3-FRB:KanMX, Tub1-mRuby2:HphMX, CUP1prLacI-GFP:HIS3, LacO:LEU2 (Chr I), *BIR1*-CEN:URA3
LY10816	Mat a, *tor1-1*, *fpr1*::NatMX, RPL13A-2xFKBP12:loxP, Bub3-FRB:KanMX, Tub1-mRuby2:HphMX, CUP1prLacI-GFP:HIS3, LacO:TRP1 (Chr VIII), *BIR1*-CEN:URA3
LY10820	Mat a, *tor1-1*, *fpr1*::NatMX, RPL13A-2xFKBP12:loxP, Bub3-FRB:KanMX, Tub1-mRuby2:HphMX, CUP1prLacI-GFP:HIS3, LacO:TRP1 (Chr X), *BIR1*-CEN:URA3
LY10821	Mat a, *tor1-1*, *fpr1*::NatMX, RPL13A-2xFKBP12:loxP, Bub3-FRB:KanMX, Tub1-mRuby2:HphMX, CUP1prLacI-GFP:HIS3, LacO:TRP1 (Chr II), *SLI15*-CEN:URA3, *BIR1*-CEN:LEU2
LY10822	Mat a, *tor1-1*, *fpr1*::NatMX, RPL13A-2xFKBP12:loxP, Bub3-FRB:KanMX, Tub1-mRuby2:HphMX, CUP1prLacI-GFP:HIS3, LacO:TRP1 (Chr VIII), *SLI15*-CEN:URA3, *BIR1*-CEN:LEU2
LY10823	Mat a, *tor1-1*, *fpr1*::NatMX, RPL13A-2xFKBP12:loxP, Bub3-FRB:KanMX, Tub1-mRuby2:HphMX, CUP1prLacI-GFP:HIS3, LacO:TRP1 (Chr X), *SLI15*-CEN:URA3, *BIR1*-CEN:LEU2
LY10824	Mat a, *tor1-1*, *fpr1*::NatMX, RPL13A-2xFKBP12:loxP, Bub3-FRB:KanMX, Tub1-mRuby2:HphMX, CUP1prLacI-GFP:HIS3, LacO:LEU2 (Chr III), *BIR1*-CEN:URA3, *SLI15*-CEN:TRP1
LY10825	Mat a, *tor1-1*, *fpr1*::NatMX, RPL13A-2xFKBP12:loxP, Bub3-FRB:KanMX, Tub1-mRuby2:HphMX, CUP1prLacI-GFP:HIS3, LacO:LEU2 (Chr I), *BIR1*-CEN:URA3, *SLI15*-CEN:TRP1
LY10874	haploid A1, wild-type, non-evolved
LY10875	haploid A1, wild-type, evolved
LY10876	haploid A4, wild-type, non-evolved
LY10877	haploid A4, wild-type, evolved
LY10878	haploid B2, wild-type, non-evolved
LY10879	haploid B2, wild-type, evolved
LY10880	haploid C1, wild-type, non-evolved
LY10881	haploid C1, wild-type, evolved
LY10882	haploid C3, wild-type, non-evolved
LY10883	haploid C3, wild-type, evolved
LY10884	haploid D1, wild-type, non-evolved

LY10885	haploid D1, wild-type, evolved
LY10886	haploid D3, wild-type, non-evolved
LY10887	haploid D3, wild-type, evolved
LY10892	haploid F3, wild-type, non-evolved
LY10893	haploid F3, wild-type, evolved
LY10894	haploid G1, wild-type, non-evolved
LY10895	haploid G1, wild-type, evolved
LY10896	haploid A2, *bub3*::LEU2, non-evolved
LY10897	haploid A2, *bub3*::LEU2, evolved
LY10898	haploid A3, *bub3*::LEU2, non-evolved
LY10899	haploid A3, *bub3*::LEU2, evolved
LY10900	haploid B3, *bub3*::LEU2, non-evolved
LY10901	haploid B3, *bub3*::LEU2, evolved
LY10902	haploid C2, *bub3*::LEU2, non-evolved
LY10903	haploid C2, *bub3*::LEU2, evolved
LY10904	haploid C4, *bub3*::LEU2, non-evolved
LY10905	haploid C4, *bub3*::LEU2, evolved
LY10906	haploid D2, *bub3*::LEU2, non-evolved
LY10907	haploid D2, *bub3*::LEU2, evolved
LY10908	haploid D4, *bub3*::LEU2, non-evolved
LY10909	haploid D4, *bub3*::LEU2, evolved
LY10910	haploid E3, *bub3*::LEU2, non-evolved
LY10911	haploid E3, *bub3*::LEU2, evolved
LY10912	haploid E4, *bub3*::LEU2, non-evolved
LY10913	haploid E4, *bub3*::LEU2, evolved
LY10914	haploid F4, *bub3*::LEU2, non-evolved
LY10915	haploid F4, *bub3*::LEU2, evolved
LY10916	haploid G4, *bub3*::LEU2, non-evolved
LY10917	haploid G4, *bub3*::LEU2, evolved
LY10918	Mat a, evolved
LY10919	Mat a, evolved, *BUB3*-CEN:URA3
LY10920	haploid C2, *bub3*::LEU2, evolved, *BUB3*-CEN:URA3
LY10921	haploid E3, *bub3*::LEU2, evolved, *BUB3*-CEN:URA3
LY10922	haploid G4, *bub3*::LEU2, evolved, *BUB3*-CEN:URA3
LY10923	Mat a, *tor1-1*, *fpr1*::NatMX, RPL13A-2xFKBP12:loxP, Bub3-FRB:HphMX, Tub1-mRuby2:URA3, CUP1prLacI-GFP:HIS3, LacO:LEU2 (Chr III), pP2H6 (Yeast Genome Tiling collection)
LY10924	Mat a, *tor1-1*, *fpr1*::NatMX, RPL13A-2xFKBP12:loxP, Bub3-FRB:HphMX, Tub1-mRuby2:URA3, CUP1prLacI-GFP:HIS3, LacO:LEU2 (Chr III), pP2A7 (Yeast Genome Tiling collection)
LY10925	Mat a, *tor1-1*, *fpr1*::NatMX, RPL13A-2xFKBP12:loxP, Bub3-FRB:HphMX, Tub1-mRuby2:URA3, CUP1prLacI-GFP:HIS3, LacO:LEU2 (Chr III), pP2F7 (Yeast Genome Tiling collection)

LY10926	Mat a, *tor1-1*, *fpr1*::NatMX, RPL13A-2xFKBP12:loxP, Bub3-FRB:HphMX, Tub1-mRuby2:URA3, CUP1prLacI-GFP:HIS3, LacO:LEU2 (Chr III), pP2H7 (Yeast Genome Tiling collection)
LY10927	Mat a, *tor1-1*, *fpr1*::NatMX, RPL13A-2xFKBP12:loxP, Bub3-FRB:HphMX, Tub1-mRuby2:URA3, CUP1prLacI-GFP:HIS3, LacO:LEU2 (Chr III), pP2B9 (Yeast Genome Tiling collection)
LY10928	Mat a, *tor1-1*, *fpr1*::NatMX, RPL13A-2xFKBP12:loxP, Bub3-FRB:HphMX, Tub1-mRuby2:URA3, CUP1prLacI-GFP:HIS3, LacO:LEU2 (Chr III), pP2D9 (Yeast Genome Tiling collection)
LY10929	Mat a, *tor1-1*, *fpr1*::NatMX, RPL13A-2xFKBP12:loxP, Bub3-FRB:HphMX, Tub1-mRuby2:URA3, CUP1prLacI-GFP:HIS3, LacO:LEU2 (Chr III), pP2G9 (Yeast Genome Tiling collection)
LY10930	Mat a, *tor1-1*, *fpr1*::NatMX, RPL13A-2xFKBP12:loxP, Bub3-FRB:HphMX, Tub1-mRuby2:URA3, CUP1prLacI-GFP:HIS3, LacO:LEU2 (Chr III), pP2C10 (Yeast Genome Tiling collection)
LY10931	Mat a, *tor1-1*, *fpr1*::NatMX, RPL13A-2xFKBP12:loxP, Bub3-FRB:HphMX, Tub1-mRuby2:URA3, CUP1prLacI-GFP:HIS3, LacO:LEU2 (Chr III), pP2D10 (Yeast Genome Tiling collection)
LY10932	Mat a, *tor1-1*, *fpr1*::NatMX, RPL13A-2xFKBP12:loxP, Bub3-FRB:HphMX, Tub1-mRuby2:URA3, CUP1prLacI-GFP:HIS3, LacO:LEU2 (Chr III), pP2G10 (Yeast Genome Tiling collection)
LY10933	Mat a, *tor1-1*, *fpr1*::NatMX, RPL13A-2xFKBP12:loxP, Bub3-FRB:HphMX, Tub1-mRuby2:URA3, CUP1prLacI-GFP:HIS3, LacO:LEU2 (Chr III), pP2A11 (Yeast Genome Tiling collection)
LY10934	Mat a, *tor1-1*, *fpr1*::NatMX, RPL13A-2xFKBP12:loxP, Bub3-FRB:HphMX, Tub1-mRuby2:URA3, CUP1prLacI-GFP:HIS3, LacO:LEU2 (Chr III), pP2G11 (Yeast Genome Tiling collection)
LY11030	Mat a, *tor1-1*, *fpr1*::NatMX, RPL13A-2xFKBP12:loxP, Bub3-FRB:KanMX, Tub1-mRuby2:HphMX, CUP1prLacI-GFP:HIS3, LacO:TRP1 (Chr II), CEN:URA3, CEN:LEU2
