# Supplementary material for: Aneuploidy of specific chromosomes is beneficial to cells lacking spindle checkpoint protein Bub3
Source: PLoS Genet. 2025 Feb 4;21(2):e1011576. doi: 10.1371/journal.pgen.1011576 (PMC11819610; doi:10.1371/journal.pgen.1011576)
Supplement: S4 Table — (PDF) [file pgen.1011576.s009.pdf]

S4_Table: Plasmid list

Plasmid number	Details	
pLB113	Cup1pr-LacI-GFP:HIS3	Lacefield lab
pLB178	BUB3pr-*BUB3*	Lacefield lab
pLB226	NDT80pr-NDT80:LEU2 (2μ)	[1]
pLB227	LEU2 (2μ)	[1]
pLB486	BIR1pr-*BIR1*:TRP1	Lacefield lab
pLB499	SLI15pr-*SLI15*:LEU2	Lacefield lab
pLB551	LacO:Leu2 (chromosome III)	Lacefield lab
pLB553	BUB3pr-*BUB3*:LEU2 (2μ)	this study
pLB568	BIK1pr-*BIK1*:TRP1 (CEN)	this study
pLB569	SLI15pr-*SLI15*:TRP1 (CEN)	this study
pLB572	LacO:TRP1 (chromosome II)	this study
pLB573	LacO:TRP1 (chromosome X)	this study
pLB586	SLI15pr-*SLI15*:URA3 (CEN)	this study
pLB587	NBL1pr-*NBL1*:URA3 (CEN)	this study
pLB589	LacO:Leu2 (chromosome I)	this study
pLB599	BUB3pr-*BUB3*:URA3 (CEN)	this study
pLB600	BIK1pr-*BIK1*:URA3 (CEN)	this study
pLB610	*BIK1pr-BIK1*:LEU2 (CEN)	this study
pLB612	CSM1pr-*CSM1*:URA3 (CEN)	this study
pLB614	KCC4-*KCC4*:URA3 (CEN)	this study
pLB615	SLI15pr-*SLI15*:LEU2 (CEN)	this study
pLB618	BIR1pr-*BIR1*:LEU2 (CEN)	this study
pLB619	BIR1pr-*BIR1*:URA3 (CEN)	this study
pV111	LEU2 (CEN)	[2]
pV112	URA3 (CEN)	[2]
pV124	TRP1 (CEN)	[2]
pV342	HIS3pr-mRuby2-Tub1:URA3	Addgene #50639
pV344	HIS3pr-mRuby2-Tub1:HphMX	Addgene #50633
Yeast Genome Tiling collection		[3]

1. Gavade JN, Puccia CM, Herod SG, Trinidad JC, Berchowitz LE, Lacefield S. Identification of 14-3-3 proteins, Polo kinase, and RNA-binding protein Pes4 as key regulators of meiotic commitment in budding yeast. Curr Biol. 2022;32: 1534-1547.e9. doi:10.1016/j.cub.2022.02.022
2. Sikorski RS, Hieter P. A system of shuttle vectors and yeast host strains designed for efficient manipulation of DNA in Saccharomyces cerevisiae. Genetics. 1989;122: 19–27. doi:10.1093/genetics/122.1.19
3. Jones GM, Stalker J, Humphray S, West A, Cox T, Rogers J, et al. A systematic library for comprehensive overexpression screens in Saccharomyces cerevisiae. Nat Methods. 2008;5: 239–241. doi:10.1038/nmeth.1181
