# Supplementary material for: Aneuploidy of specific chromosomes is beneficial to cells lacking spindle checkpoint protein Bub3
Source: PLoS Genet. 2025 Feb 4;21(2):e1011576. doi: 10.1371/journal.pgen.1011576 (PMC11819610; doi:10.1371/journal.pgen.1011576)
Supplement: S5 Table — (PDF) [file pgen.1011576.s010.pdf]

**S5_Table: Primer list**

| Primer number | Primer name | Sequence |
|---|---|---|
| LO3531 | Bik1_**BamHI**_Fw | ATATAT**ggatcc**ATGAGTGTGTCATCACTGTGG |
| LO3532 | Bik1_**HindIII**_Rev | ATATAT**aagctt**GACAAAGCCACCAATGGAACT |
| LO3136 | Sli15_**XhoI**_Fw | CTAGTG**ctcgag**GCAATCTCATTCAGCAGGTC |
| LO3137 | Sli15_**XbaI**_Rev | ATACGT**tctaga**CATGGAAACAAAGGCAGGTG |
| LO3499 | Nbl1_**SalI**_Fw | ATATAT**gtcgac**CCAGCAAGAATCTTCCCAAAC |
| LO3500 | Nbl1_**SacI**_Rev | ATATAT**gagctc**TTGTCTTCAGCGGCCACATA |
| LO3455 | Bub3_**BamHI**_Fw | ATATAT**ggatcc**GACACCCATTGGCGAATCCTC |
| LO3456 | Bub3_**HindIII**_Rev | ATATAT**aagctt**GATCGCCAAGACCTAAGTGGG |
| LO3555 | Csm1_**SalI**_Fw | ATATAT**gtcgac**GGGTAAATTAGGGCTTTCCTGG |
| LO3530 | Csm1_**SacI**_Rev | ATATAT**gagctc**CGTTCAACTGTGAGGTGTGT |
| LO3680 | Kcc4_**XhoI**_Fw | ATATAT**ctcgag**GGAGAATGCACACCTTCGTA |
| LO3681 | Kcc4_**XbaI**_Rev | ATATAT**tctaga**TGGGGATCGATTATCCCTCC |
| LO3481 | Bir1_**SalI**_Fw | ATATAT**gtcgac**GTTTCCTTCTGTTAGTGCAGAGTC |
| LO3482 | Bir1_**SalI**_Rev | ATATAT**gagctc**GACGAATCAATGCCTGACACT |
