# Supplementary material for: Aneuploidy of specific chromosomes is beneficial to cells lacking spindle checkpoint protein Bub3
Source: PLoS Genet. 2025 Feb 4;21(2):e1011576. doi: 10.1371/journal.pgen.1011576 (PMC11819610; doi:10.1371/journal.pgen.1011576)
Supplement: S6 Table — (PDF) [file pgen.1011576.s011.pdf]

S6_Table: Reagent list

Reagent	Source	Catalog number
Yeast extract	Gibco	212720
Peptone	Gibco	211677
Dextrose (D-Glucose) anhydrous	Fisher chemical	D16-10
Potassium acetate	Fisher chemical	P171-500
BD Difco™Yeast nitrogen base without amino acids	Thermo Fisher Scientific	291920
Sodium chloride	EMD Millipore	SX0420-5
Tryptone (Casein Peptone)	Sunrise Science Products	1914-1KG
Bacto agar	Sunrise Science Products	1910-5KG
Rapamycin	Thermo Fisher Scientific	AAJ62473MF
Alpha factor	Zymo Research	Y1001
G418 sulphate	Fisher Bioreagents	BP673-5
Hygromycin	invitrogen	10687010
Dimethyl sulfoxide	Sigma-aldrich	5879-500ML
Ampicillin sodium salt	Sigma-aldrich	A9518-5G
Kanamycin sulfate	Sigma-aldrich	60615-5G
Glycerol	invitrogen	15514029
Phenol/chloroform/isoamyl alcohol	Fisher Bioreagents	BP1752I-400
Lithium acetate dihydrate	Sigma-aldrich	L6883-1KG
single-stranded salmon sperm DNA	Sigma-aldrich	D1626-5G
Polyethylene glycol (MW 3350)	Spectrum chemical MFG corp	25322-68-3
Benomyl	Chem Service	N11138
